# Combinatorial biosynthesis yields novel hybrid argimycin P alkaloids with diverse scaffolds in *Streptomyces argillaceus*

**DOI:** 10.1111/1751-7915.14167

**Published:** 2022-11-08

**Authors:** Suhui Ye, Giovanni Ballin, Ignacio Pérez‐Victoria, Alfredo F. Braña, Jesús Martín, Fernando Reyes, José A. Salas, Carmen Méndez

**Affiliations:** ^1^ Departamento de Biología Funcional e Instituto Universitario de Oncología del Principado de Asturias (I.U.O.P.A) Universidad de Oviedo Oviedo Spain; ^2^ Instituto de Investigación Sanitaria de Asturias (ISPA) Oviedo Spain; ^3^ Fundación MEDINA Centro de Excelencia en Investigación de Medicamentos Innovadores en Andalucía Armilla, Granada Spain

## Abstract

Coelimycin P1 and argimycins P are two types of polyketide alkaloids produced by *Streptomyces coelicolor* and *Streptomyces argillaceus*, respectively. Their biosynthesis pathways share some early steps that render very similar aminated polyketide chains, diverging the pathways afterwards. By expressing the putative isomerase *cpkE* and/or the putative epoxidase/dehydrogenase *cpkD* from the coelimycin P1 gene cluster into *S. argillaceus* wild type and in argimycin mutant strains, five novel hybrid argimycins were generated. Chemical characterization of those compounds revealed that four of them show unprecedented scaffolds (quinolizidine and pyranopyridine) never found before in the argimycin family of compounds. One of these compounds (argimycin DM104) shows improved antibiotic activity. Noticeable, biosynthesis of these quinolizidine argimycins results from a hybrid pathway created by combining enzymes from two different pathways, which utilizes an aminated polyketide chain as precursor instead of lysine as it occurs for other quinolizidines.

## Graphical abstract

Coelimycin P1 and argimycins P are two polyketide alkaloids produced by *Streptomyces coelicolor* and *Streptomyces argillaceus* respectively, whose biosynthesis pathways share some early steps. By expressing *cpkD* or *cpkDE* genes from later biosynthetic steps of coelimycin P1 into *S. argillaceus* strains, five novel hybrid argimycins were generated with unprecedented scaffolds, one of which with improved antibiotic activity.

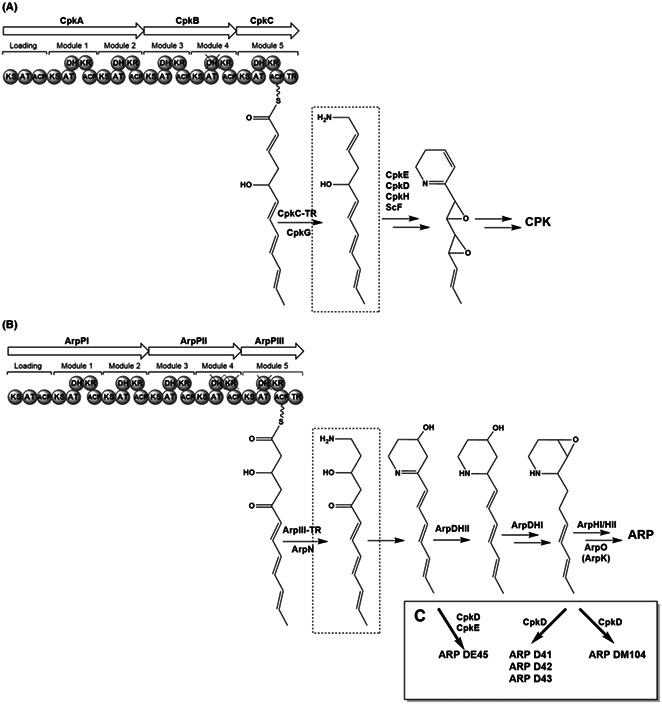

## INTRODUCTION

**FIGURE 1:**
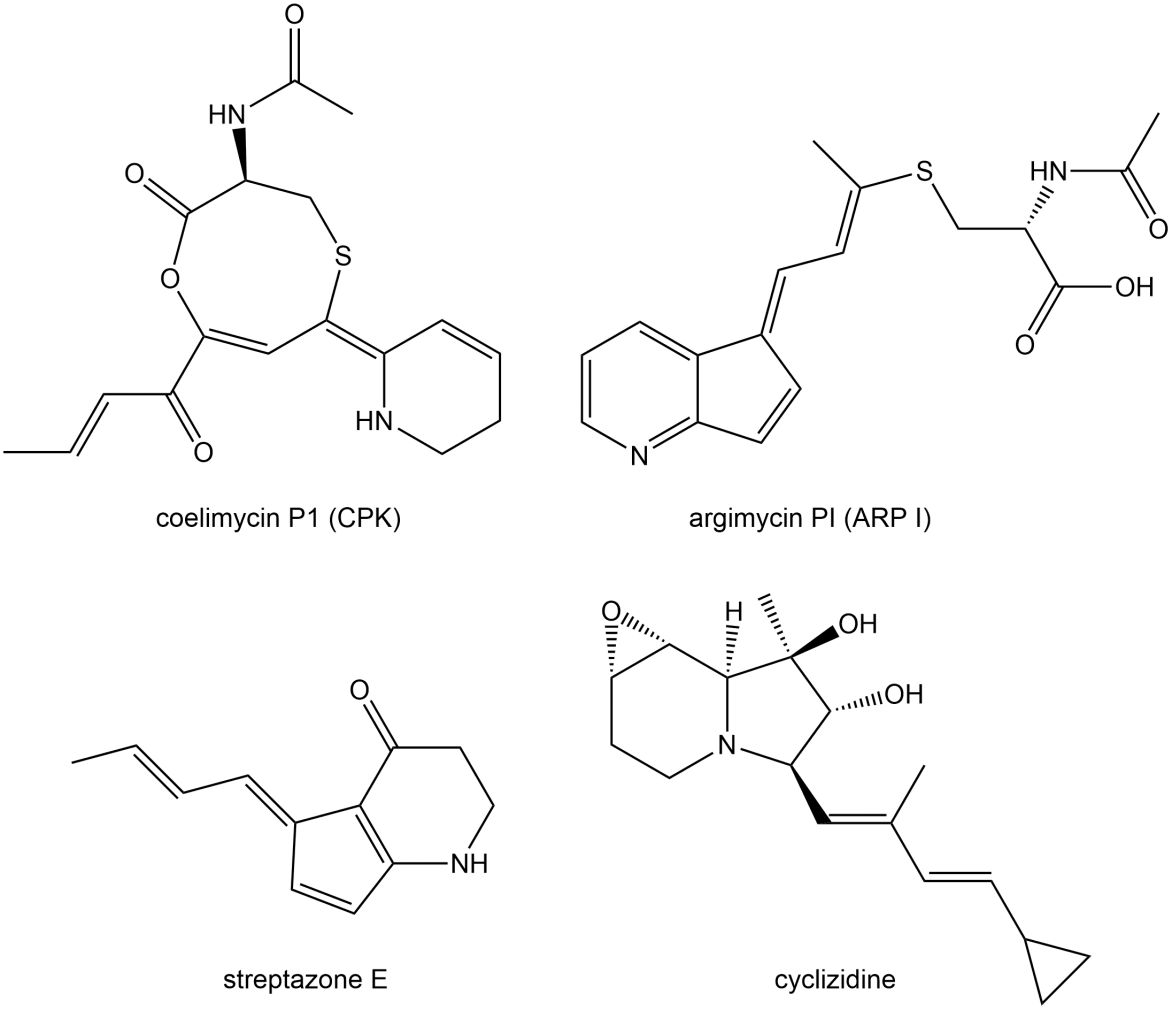
Structures of some piperidine‐containing polyketide alkaloids from *Streptomyces*.

Alkaloids are a group of complex nitrogen‐containing natural products derived from a variety of sources, including bacteria, fungi, insects, plants, and animals (Cushnie et al., 2014; Rathbone & Bruce, 2002; Sigrist et al., 2015). They have attracted a lot of interest due to their multiple biological activities, such as analgesics, antibiotic, antibacterial‐enhancing, antivirulence, antifungal, anticancer, anti‐inflammatory, or antiplatelet (Ain et al., 2016; Cushnie et al., 2014; Khan et al., 2017; Mondal et al., 2019; Peng et al., 2019; Rathbone & Bruce, 2002). Currently, more than 27,000 alkaloids have been identified from natural sources that according to their structures can be divided into non‐heterocyclic (or atypical/protoalkaloids) and heterocyclic (or typical) alkaloids (Cushnie et al., 2014). Despite the abundant diversity of typical alkaloids, their basic units are limited and only around 20 different rings have been identified so far, being the two most frequently found the indolizidine and piperidine rings (Cushnie et al., 2014; Peng et al., 2016). Most piperidine‐containing alkaloids or true alkaloids derive from amino acids, but the so‐called pseudoalkaloids have a polyketide origin and acquire their nitrogen atoms via transamination reactions (Awodi et al., 2017; Sigrist et al., 2015) (Figure 1).

These polyketides are synthesized by Type I polyketide synthases (PKSs), which are multifunctional enzymes organized into modules. Each module is responsible for one elongation step and contains different domains in which specific enzymatic reactions occur. They contain three essential domains that collaborate to produce a β‐keto ester intermediate: a β‐ketoacyl synthase (KS), an acyltransferase (AT), and an acyl carrier protein (ACP). In addition, those modules may contain optional domains such as a β‐ketoreductase (KR), a dehydratase (DH), and an enoyl reductase (ER), which can act on the resultant β‐keto group originating hydroxy groups, double bonds or methylene groups at different locations of the molecule, thus generating a variety of polyketide chains (Risdian et al., 2019).

Actinobacteria are the most prolific microbial source of natural products, which show diverse chemical structures and complexity (Katz & Baltz, 2016). Several indolizidine and piperidine polyketide alkaloids have been isolated from actinomycetes such as osmanicin (Samy et al., 2019), strepchazolins (Yang et al., 2017), chartrenoline (Liu et al., 2019), cyclizidines (Freer et al., 1982; Jiang et al., 2018), streptopiridines (Groenhagen et al., 2014), coelimycin P1 (Gomez‐Escribano et al., 2012), streptazones (Liu et al., 2013; Puder et al., 2000), or argimycins P (Ye et al., 2017). However, to date, only the biosynthesis pathway for cyclizidine (Huang et al., 2015; Peng et al., 2016), coelimycin P1 (Awodi et al., 2017; Gomez‐Escribano et al., 2012), streptazone E (Ohno et al., 2015) and argimycins P (Ye et al., 2017, 2018) have been partially or totally characterized. These biosynthesis pathways share some common initial steps. They start by synthesizing a polyketide chain by a type I PKS. This chain is released from the PKS as an aldehyde by its thioester reductase (TR) domain, and subsequently reductively aminated by a ω‐transaminase (Awodi et al., 2017; Peng et al., 2016). The resultant intermediate may further proceed through different steps such as cyclization and/or modifications by other tailoring enzymes.

**FIGURE 2:**
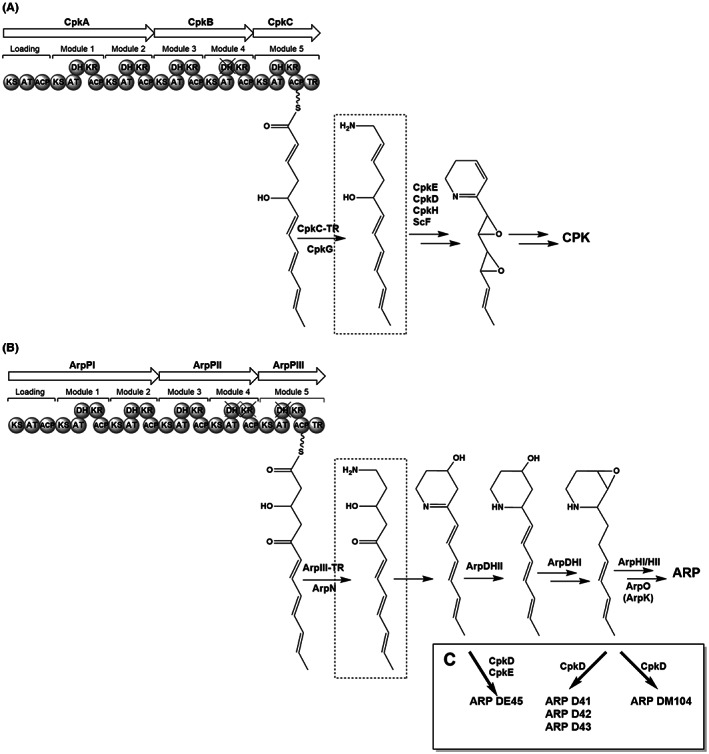
Analysis of PKS modules and biosynthesis steps of coelimycin P1 and argimycins P. (A) coelimycin P1, (B) argimycins P and (C) proposed biosynthesis of hybrid argimycin compounds. ARP, argimycins P; CPK, coelimycin P1.

Coelimycin P1 (CPK) and argimycins P (ARP) (Figure 1) are two polyketide alkaloids encoded by the two cryptic biosynthesis gene clusters (BGCs) *cpk* and *arp* from *S. coelicolor* and *S. argillaceus*, respectively (Gomez‐Escribano et al., 2012; Ye et al., 2017). They contain genes encoding a type I PKS, which synthesize polyketide chains of the same length. After being released, these carbon chains suffer a transamination by CpkG and ArpN respectively, as it has been shown in vitro for CPK, and in vivo for ARP (Awodi et al., 2017; Ye et al., 2017) (Figure 2). Afterwards both pathways diverge. It has been proposed that the biosynthesis pathway for CPK might proceed through a series of oxidations, a cyclodehydration, and the addition of N‐acetylcysteine to give rise the final product coelimycin P1 (Gomez‐Escribano et al., 2012). On the other hand, by characterizing the metabolite profiles of mutants in different *arp* genes it was shown that the biosynthesis of ARP proceeds through the formation of a piperideine ring and reduction of its imine group, cyclization to give rise to the five‐membered ring, aminoxidation and attachment of a N‐acetylcysteine residue (Ye et al., 2017, 2018).

In this work, we show that by expressing selected *cpk* genes from *S. coelicolor* involved in tailoring modifications of CPK, into *S. argillaceus* wild type and *arp* mutant strains, novel hybrid ARP compounds were generated, some of which constitute novel scaffolds with improved antibiotic activity.

## EXPERIMENTAL PROCEDURES

### Strains, culture conditions, plasmids and DNA manipulations

*Streptomyces coelicolor* M145 (Kieser et al., 2000) was used as source of DNA to amplify *cpk* genes. *S. argillaceus* ATCC 12956 and *S. argillaceus* MARPPIII, MARPDHI, MARPHI, MARPHII and MARPO mutant strains (Ye et al., 2017, 2018) were used as hosts to express *cpk* genes. *Escherichia coli* DH10B (Invitrogen) and *E. coli* ET12567/pUB307 (Kieser et al., 2000) were used as cloning hosts for plasmid propagation and for conjugation experiments, respectively. MA and SM10 media (Fernández et al., 1998; Ye et al., 2017) were used for sporulation and ARP production, respectively. When required, antibiotics were added to media at the following final concentrations: kanamycin (50 μg/ml), nalidixic acid (25 μg/ml), apramycin (25 μg/ml), and thiostrepton (50 μg/ml). Plasmid pCR‐Blunt (Invitrogen) was used for subcloning. Plasmid pSETEc and pSETETc (Cano‐Prieto et al., 2015) were used to express *cpk* genes in *S. argillaceus* strains. DNA manipulations, intergeneric conjugations and transformations were carried out according to standard procedures for *Streptomyces* (Kieser et al., 2000) and for *E. coli* (Sambrook & Russell, 2001). PCR amplifications were carried out using Herculase II (Stratagene) and 5% dimethyl‐sulphoxide (DMSO). Purified amplicons were sequenced, and sequences were compared with others in databases. Sequence analysis was carried out using BLAST (Altschul et al., 1997). Bioassays were performed as previously described (Vilches et al., 1990).

### Plasmid constructs for expressing *cpk* genes

Several plasmids were constructed to express *cpk* genes from *S. coelicolor* into *S. argillaceus* (Table 1). To this aim, genes were amplified and subcloned under the control of an erythromycin resistance promoter (see Supporting Information).

**TABLE 1:** Plasmids and *Streptomyces argillaceus* recombinant strains generated in this work

Plasmid	Gene	Recombinant strain
pSETEc	–	WT‐pSETEc MARPPIII‐pSETEc
pSETEcScF	*scF*	WT‐*scF*
pSETEcCpkH	*cpkH*	WT‐*cpkH*
pSETEcCpkD	*cpkD*	WT‐*cpkD* MARPPIII‐*cpkD*
pSETEcCpkE	*cpkE*	WT‐*cpkE* MARPPIII‐*cpkE*
pSETEcCpkDE	*cpkD + cpkE*	WT‐*cpkDE* MARPPIII‐*cpkDE*
pSETETc	*–*	MARPDHI‐pSETETc MARPHI‐pSETETc MARPO‐pSETETc
pSETETcCpkD	*cpkD*	MARPDHI‐*cpkD* MARPHI‐*cpkD* MARPO‐*cpkD*
pSETETcCpkDE	*cpkD + cpkE*	MARPDHI‐*cpkDE* MARPHI‐*cpkDE* MARPO‐*cpkDE*

### UPLC analysis and purification of argimycin derivatives

Argimycins P derivatives were extracted with n‐butanol and preliminary analyses were carried out as previously reported (Ye et al., 2017). Further analysis of cultures of *cpkD, cpkE* and *cpkDE* expressing strains were carried out using a different column, an HSS T3 column (1.8 μm, 2.1 × 100 mm; Waters), with mixtures of acetonitrile and 0.1% trifluoroacetic acid as mobile phase. Samples were eluted with pure 0.1% trifluoroacetic acid for 1 min, followed by a linear gradient from 0% to 60% acetonitrile in 7 min, at a flow rate of 0.5 ml/min and a column temperature of 35°C. Detection and spectral characterization was performed by photodiode array detection and Empower software (Waters). Chromatograms were extracted at 300 and 280 nm.

For purification purposes, strains were grown by a two‐step culture method, as previously described (Fernández et al., 1998), using forty 250‐mililitre Erlenmeyer flasks in the production step. Purification of ARP novel compounds was carried out as previously described (Ye et al., 2017), but using an Atlantis T3 column (3 μm, 2.1 × 150 mm; Waters) and using isocratic chromatography conditions optimized for each compound.

### Structural elucidation

Structural elucidation of each compound was carried out using a combination of ESI‐TOF mass spectrometry and NMR spectroscopy (see Supporting Information). HRMS spectra were collected from LC‐DAD‐MS analyses using an Agilent 1200 Rapid Resolution HPLC system equipped with a SB‐C8 column (2.1 × 30 mm, Zorbax) and coupled to a Bruker maXis mass spectrometer. Chromatographic and ionization conditions were identical to those previously described (Martín et al., 2014; Pérez‐Victoria et al., 2016). UV/vis (DAD) spectra were also collected in the same chromatographic analyses. NMR spectra were recorded in CD_3_OD at 24°C on a Bruker AVANCE III‐500 (500 MHz and 125 MHz for ^1^H and ^13^C NMR, respectively) equipped with a 1.7 mm TCI MicroCryoProbe™, using the residual solvent signal as internal reference (δ_H_ 3.32 and δ_C_ 47.5). The molecular formula obtained from the experimental accurate mass of each compound alongside the analysis of the 1D and 2D NMR spectra rendered the full connectivity and relative stereochemistry of the compounds.

## RESULTS

### Comparative analysis between coelimycin P1 and argimycins P biosynthesis pathways

A comparative analysis between *cpk* and *arp* reveals only five structural genes in common: the ones encoding a type I PKS (*cpkA, cpkB, cpkC* and *arpPI, arpPII, arpPIII*), the aminotransferase (*cpkG* and *arpN*), and a type II thioesterase (*scoT* and *arpT*) (Figure S1). Both PKSs are quite similar (Figure 2); they consist of three subunits with a similar module and domain organization. They only differ at modules 4 and 5: module 4 from CpkB contains a KR domain that is inactive in ArpPII; and module 5 in CpkC contains a DH domain that is inactive in ArpPIII (Figure 2) (Pawlik et al., 2007; Ye et al., 2017). Therefore, these PKSs synthesize polyketide chains of the same length that differ at C‐2, C‐3 and C‐5, which are aminated after being released from those PKSs (Figure 2). However, afterwards both pathways follow different routes that will involve different and specific enzymatic reactions in each pathway. Thus, the *cpk* BGC contains three genes (*scF*, *cpkH* and *cpkD*) that encode proteins similar to known Flavin‐dependent epoxidases/dehydrogenases, which have been proposed as candidates to catalyse the two epoxidations and/or the oxidation of a hydroxy group of CPK biosynthesis intermediates (Gomez‐Escribano et al., 2012). Since those gene functions are absent in the *arp* BGC, we hypothesized that expression of some of those genes in *S. argillaceus* could generate hybrid ARP compounds. Therefore, we selected those genes (as well as *cpkE* that encodes a possible isomerase) for being expressed into *S. argillaceus*. The reason why *cpkE* was included was that its coding region overlaps with that of *cpkD*, which suggests a functional relationship between them.

### Heterologous expression of *cpk* genes in *S. argillaceus* wild type strain

*scF, cpkH, cpkD, cpkE* and *cpkDE* genes were PCR amplified from *S. coelicolor* M145 and independently cloned under the control of the erythromycin resistance promoter (*ermEp*) in the integrative plasmid pSETEc, as described in Supporting Information (Table 1).

The respective constructs (as well as the empty vector) were independently introduced into *S. argillaceus* wild type strain (WT), and the metabolite profiles of the resultant recombinant strains analysed by UPLC. As control, the different constructs were also expressed into MARPPIII, a mutant in the *arpPIII* PKS gene that is blocked in the biosynthesis of the ARP polyketide chain (Ye et al., 2017). Metabolite profiles produced by MARPPIII recombinant strains were used to discriminate differential peaks not related to ARP.

**FIGURE 3:**
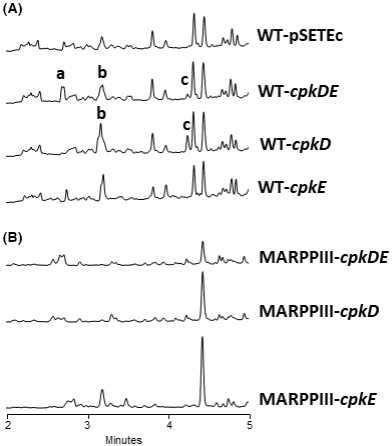
UPLC chromatograms at 300 nm of butanol extracts of *S. argillaceus* strains expressing *cpk* genes. (A) *S. argillaceus* wild type strain (WT) and (B) *S. argillaceus* MARPPIII. Peaks a to c contain novel argimycins (ARP) compounds identified in this work: ARP DE45 (peak a), ARP D43 and ARP D44 (peak b) and ARP D41 and ARP D42 (peak c).

Expression of either *scF* or *cpkH* into *S. argillaceus* WT did not result in production of any detectable new compound (data not shown). However, when either *cpkD* or *cpkDE* was expressed in *S. argillaceus* WT, some differential peaks were detected at 300 nm that were absent in the control strain containing the empty vector (Figure 3A). Peak c was detected in both strains WT‐*cpkD* and WT‐*cpkDE*, while peak a was only observed in WT‐*cpkDE*. Expression of *cpkE* alone did not result in production of neither of these peaks (Figure 3A). These two peaks were not produced when either *cpkD* or *cpkDE* were expressed in MARPPIII mutant (MARPPIII‐*cpkD* and MARPPIII‐*cpkDE*; Figure 3B), which indicates that compounds in peaks a and c were ARP derivatives. These results suggest formation of novel hybrid ARP compounds, and show that formation of compounds in peak c only requires expression of *cpkD*, while both CpkD and CpkE are necessary for production of compounds in peak a, indicating that CpkD would act before than CpkE. In addition, a careful analysis of compounds in peak b from WT‐*cpkD* and WT‐*cpkDE* revealed they contain a new compound with a similar absorption spectrum to those compounds in peak c. This newly identified compound was absent in the control strain (WT‐pSETEc), in WT‐*cpkE*, and in all MARPPIII recombinant strains (Figure 3), indicating it was a hybrid ARP compound.

### Heterologous expression of *cpkD* , *cpkE* and *cpkDE* in *S. argillaceus* mutant strains

In light of the positive results obtained above by expressing *cpkD* and *cpkDE* in *S. argillaceus* WT strain, and taking advantage of the existence of a collection of knockout mutant strains in different structural genes of *arp* BGC (Ye et al., 2018), we attempted to express those genes in some of these mutant strains. This had two purposes: (i) to generate new ARP derivatives resulting from the enzymatic activity of Cpk proteins on compounds accumulated by those mutant strains and (ii) to determine the contribution of *arp* gene products to the formation of the different new hybrid compounds. We selected *S. argillaceus* MARPDHI, *S. argillaceus* MARPHI, *S. argillaceus* MARPHII and *S. argillaceus* MARPO, which are mutants in specific steps of ARP biosynthesis that accumulate different biosynthesis intermediates (Ye et al., 2018). To express *cpkD* and *cpkDE* in these mutants, it was necessary to generate two new constructs (pSETETcCpkD and pSETETcCpkDE) using as a vector pSETETc that confers resistance to thiostrepton, since the constructs used before (pSETEcCpkD and pSETEcCpkDE) confer resistance to apramycin and the *S. argillaceus* mutants already display resistance to this antibiotic (Ye et al., 2018).

**FIGURE 4:**
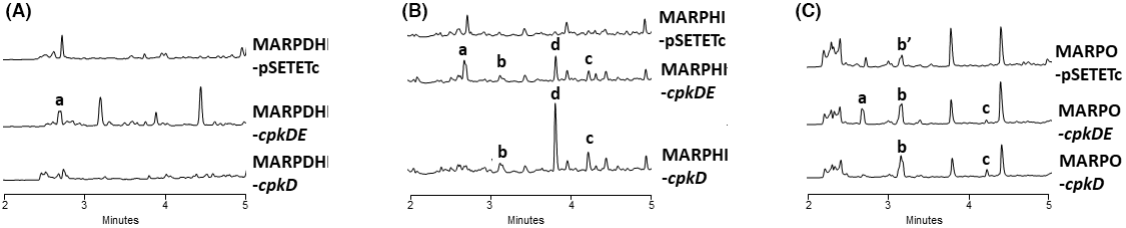
UPLC chromatograms at 300 nm of butanol extracts of *S. argillaceus* mutant strains expressing *cpk* genes. (A) *S. argillaceus* MARPDHI, (B) *S. argillaceus* MARPHI and (C) *S. argillaceus* MARPO. Peak b’ corresponds to ARP D44 and peak d to ARP DM104.

MARPDHI is a knockout mutant strain in *arpDHI*. This gene encodes a putative acyl‐CoA dehydrogenase, proposed to oxidize a biosynthesis intermediate at an early step of ARP biosynthesis, before the five‐membered ring formation (Ye et al., 2018). When *cpkD* or *cpkDE* were expressed into MARPDHI, the resultant strains MARPDHI‐*cpkD* and MARPDHI‐*cpkDE* did not produce any of those ARP derivatives contained in peaks b and c (Figure 4A). This indicates that production of hybrid ARPs in those peaks requires expression of *arpDHI*. However, peak a could be detected in chromatograms from MARPDHI‐*cpkDE* (Figure 4A), which indicates that production of this peak is independent of expression of *arpDHI*.

MARPHI and MARPHII are knockout *S. argillaceus* mutants in *arpHI* and *arpHII*, respectively. These genes encode putative cyclases that are essential for the formation of the five‐membered ring of bicyclic ARP (Ye et al., 2018). Previous studies have shown that both mutant strains produce the same metabolite profiles and accumulate the same ARP compounds (Ye et al., 2018). Likewise, expression of *cpkD* and *cpkDE* genes in MARPHI and MAPRHII led to the production of the same compounds in both strains. Here for simplification we only show chromatograms corresponding to MARPHI recombinant strains. All peaks previously identified in *S. argillaceus* WT either expressing *cpkD* or *cpkDE* (peaks a, b and c; Figure 3A) were also detected in the corresponding MARPHI‐*cpkD* and MARPHI‐*cpkDE* strains (Figure 4B), indicating that neither ArpHI nor ArpHII enzymatic activities were essential for their biosynthesis. Additionally, a new peak (peak d) was identified at 280 nm UV/vis spectrum in MARPHI‐*cpkD* and MARPHI‐*cpkDE*, which was absent in the control strain (Figure 4B).

**FIGURE 5:**
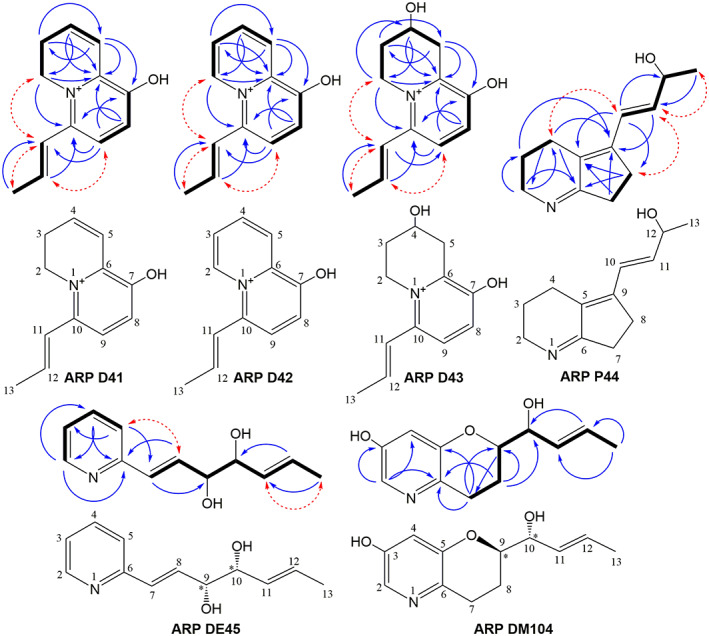
Structural elucidation of novel argimycin compounds. Structures and key COSY (bold bonds), HMBC (blue arrows) and NOESY (dashed red arrows) correlations used to determine the chemical structure of the novel argimycin P (ARP) derivatives identified in this work.

MARPO is a knockout mutant in *arpO*, which encodes a putative oxygenase necessary for the biosynthesis of ARP PI and ARP PII (Ye et al., 2018). All compounds identified in peaks a to c were also identified in the MARPO strains expressing *cpkD* and *cpkDE* (Figure 4C), indicating that ArpO enzymatic activity is likewise not necessary for their biosynthesis. No other additional ARP compound was identified. Interestingly, MARPO containing the empty vector shows a peak (peak b’ in Figure 4C) with a UV/vis spectrum with a maximum at 302 nm. A mass spectrometry analysis of this peak revealed the same protonated adduct (192 [M + H]^+^) as ARP D44 (Figure 5; see below), suggesting that albeit being a new ARP derivative identified in this work, ARP D44 does not result from the enzymatic activity of CpkD on ARP biosynthesis pathway, and therefore is not a hybrid compound.

### Purification, structural elucidation and bioactivity of novel argimycins

Compounds from peaks a to d were purified and chemically characterized by MS and NMR (see Supporting Information). Compound from peak a was purified from *S. argillaceus* WT‐*cpkDE* and was named ARP DE45. Compounds from peak c were purified from *S. argillaceus* WT‐*cpkD*. This peak contained two different compounds, ARP D41 and ARP D42, which were purified by HPLC and independently characterized. Peak b contains one major compound, ARP D43, and two minor ones, whose separation proved to be impossible in all conditions tested. The structure of one of the minor compounds, ARP D44, could also be determined. These compounds were purified from two strains, *S. argillaceus* WT‐*cpkD* and *S. argillaceus* WT‐*cpkDE*, what confirmed that compound composition of both peaks b was similar in both strains. The compound contained in peak d was purified from MARPHI‐*cpkD* and was named ARP DM104. The purification procedure afforded ARP D41 (1.5 mg), ARP D42 (1.3 mg), ARP D43 (4.1 mg), ARP D44 (1.9 mg), ARP DE45 (0.9 mg) and ARP DM104 (0.8 mg).

ARP D41 was assigned the molecular formula C_12_H_14_NO^+^ based on the observed M^+^ ion at *m/z* = 188.1076 (calcd. For C_12_H_14_NO^+^ = 188.1070, Δ = 3.2 ppm) which is in the range of that observed for argimycins P encoded by the cryptic gene cluster *arp* of *S. argillaceus* (Ye et al., 2017, 2018) and indicated seven degrees of unsaturation. Analysis of the ^1^H and HSQC NMR spectra revealed a total of 13 non‐exchangeable hydrogens. A further experiment was carried out to unequivocally confirm the number of exchangeable hydrogens by HRMS analysis. The NMR sample, prepared in CD_3_OD and thus displaying D/H exchange of the exchangeable hydrogens in the molecule, was diluted with the same solvent, and analysed by HRMS employing direct infusion. The new M^+^ ion was observed at *m/z* = 189.1145 corresponding to C_12_H_13_DNO^+^ (calcd. For C_12_H_13_DNO^+^ = 189.1133, Δ = 6.3 ppm) confirming the presence of only one exchangeable hydrogen in the compound. The HSQC spectrum revealed the presence of five *sp* ^*2*^ methines, two aliphatic methylenes (one likely bound to nitrogen), and one aliphatic methyl group. The key correlations observed in the COSY spectrum identified the different spin systems, which were connected via the long‐range correlations observed in the HMBC spectrum (Figure 5), which likewise was essential to unambiguously determine the position of the hydroxy group to finally determine the connectivity of the compound. Key NOESY correlations (Figure 5) provided further evidence of the structure and, alongside the coupling constants observed (*J*_H11‐H12_ = 15.4 Hz), allowed determining the *E* stereochemistry of the double bond in the exocyclic chain. The compound is positively charged due to the presence of a quaternized nitrogen and displays the expected chemical shifts for such a dihydroquinozilinium structure. The trifluoracetate counter ion (derived from the use of trifluoroacetic acid in the chromatographic purification) must be present in the isolated compound but it has no observable signals by ^1^H NMR. The similarity of the UV–vis (DAD) spectrum with that reported for 3,4‐dihydroquinozilinium iodide (Boekerheide & Gall, 1954) further corroborated the determined structure.

The UV–vis (DAD) spectrum of ARP D42 was very similar to that obtained for ARP D41, suggesting their structural relationship and a similar pattern of conjugation. Assuming a cationic form of the molecule with a quaternized nitrogen, the compound was assigned the molecular formula C_12_H_12_NO^+^ based on the observed M^+^ ion at *m/z* = 186.0920 (calcd. For C_12_H_12_NO^+^ = 186.0913, Δ = 3.7 ppm), indicating eight degrees of unsaturation. A deuterium exchange experiment again confirmed that the molecule contained just one exchangeable hydrogen due to the M^+^ ion observed at *m/z* = 187.0989 (calcd. For C_12_H_11_DNO^+^ = 187.0976, Δ = 6.9 ppm). Comparing its ^1^H and HSQC NMR spectra with those of ARP D41, it was clear the disappearance of the two *sp* ^*3*^ methylenes, replaced by signals corresponding to two aromatic hydrogens, suggesting the presence of a quinozilinium core rather than the dihydroquinozilinium present in ARP D41. Key correlations observed in its COSY and HMBC spectra (Figure 5) confirmed that, as expected, the compound was identical to ARP D41 but carrying an extra double bond that turns aromatic the second ring. Likewise, again key NOESY correlations (Figure 5) provided further evidence of the structure and, alongside the *J*_H11‐H12_ coupling constant of 15.4 Hz observed, allowed determining the configuration of the double bond in the exocyclic chain as *E*. The compound is positively charged and displays the expected NMR chemical shifts and UV–vis (DAD) spectrum for such a quinozilinium structure (Boekerheide & Gall, 1954).

The UV–vis (DAD) spectrum of ARP D43 was similar to that obtained for ARP D41 and ARP D42, suggesting their structural relationship and a similar pattern of conjugation. Again, assuming a cationic form of the molecule with a quaternized nitrogen, the compound was assigned the molecular formula C_12_H_16_NO_2_^+^ based on the observed M^+^ ion at *m/z* = 206.1181 (calcd. For C_12_H_16_NO_2_^+^ = 206.1176, Δ = 2.4 ppm), indicating six degrees of unsaturation. Its ^1^H NMR spectrum showed various signals very similar to those observed in ARP D41 and ARP D42, confirming their structural relationship. Integration of the spectrum revealed 14 non‐exchangeable hydrogens, indicating that—according to the molecular formula—two hydrogens were exchangeable and likely corresponded to hydroxy or phenolic groups. Additional analysis of the HSQC spectrum indicated a total of four *sp* ^*2*^ protons (two of them of aromatic nature), three aliphatic methylene groups (one of them bound to nitrogen), one aliphatic oxygenated methine and an allylic methyl. This information, together with the determined molecular formula and comparisons with ARP D41 were in agreement with a common backbone but losing a double bond of the non‐aromatic ring and gaining a hydroxy substituent in such cycle. The observed key COSY and HMBC correlations (Figure 5) confirmed the backbone connectivity and unambiguously determined the position of the new hydroxy group. Likewise, the key NOESY correlations provided further evidence on the structure and, alongside the *J*_H11‐H12_ coupling constant of 15.4 Hz observed, again stablished the *E* configuration of the double bond in the exocyclic chain. The absolute configuration of the single hydroxylated methine chiral center was not determined. The compound is positively charged and displays the expected chemical shifts and UV–vis (DAD) spectrum for such a tetrahydroquinozilinium structure (Boekerheide & Gall, 1954).

ARP D44 appeared as a minor component in a preparative HPLC fraction containing ARP D43 as main component. Its molecular formula could be stablished as C_12_H_17_NO^+^ based on the observed ion [M + H]^+^ at *m/z* = 192.1383 (calcd. For C_12_H_18_NO^+^ = 192.1383, Δm = 0 ppm), indicating five degrees of unsaturation. Fortunately, the NMR signals of this minor component did not overlap with the signals of ARP D43 (main component in the sample) and were intense enough for enabling structural elucidation by 2D NMR. The ^1^H and HSQC NMR spectra revealed that this minor component contained two olefinic hydrogens, one oxygenated methine, five aliphatic methylenes (one bound to nitrogen) and an aliphatic methyl instead of the vinyl methyl found in the previously described compounds. The different spin systems were determined from the cross‐peaks observed in its COSY spectrum and the long‐range correlations observed in the HMBC spectrum were employed to connect those and establish its final connectivity (Figure 5). The *E* configuration of the double bond in the lateral chain is based on the *J*_H10‐H11_ large coupling constant observed between the two olefinic hydrogens (15.7 Hz). Key NOESY correlations (Figure 5) provided further evidence for the structure proposed. The absolute configuration of the single hydroxylated methine chiral center was not determined.

ARP DE45 was assigned a molecular formula of C_12_H_15_NO_2_ based on the observed ion [M + H]^+^ at *m/z* = 206.1177 (calcd. For C_12_H_16_NO_2_^+^ = 206.1176, Δ = 0.5 ppm), indicating six degrees of unsaturation. Its ^1^H and HSQC NMR spectra revealed four aromatic, pyridine‐like, hydrogens plus four olefinic hydrogens. Two oxygenated methine groups were observed and also an aliphatic methyl (in vinylic position). Two spins systems were identified in the COSY spectrum (Figure 5). One corresponds to a monosubstituted pyridine (in the carbon contiguous to the nitrogen) and the other to the hydroxylated aliphatic chain substituent. The key long‐range correlations observed in the HMBC spectrum (Figure 5) confirmed this connectivity. The configuration of the double bonds was established based on the observed coupling constants (J_H7‐H8_ = 16 Hz, J_H11‐H12_ = 15.3 Hz) and key NOESY correlations (Figure 5). The target compound thus shares the same backbone as nigrifactin (Terashima et al., 1969). Not surprisingly, its UV–vis (DAD) spectrum also showed strong similarity with that of 2‐vinylpyridine in acidic methanol (Akito et al., 1993). The relative configuration of the vicinal diol moiety was stablished as *syn* (9R*, 10R*) by comparison of the coupling constants of H9 with those reported for the equivalent hydrogens in the *syn* and *anti* diastereomers of the analogous synthetic (1 *E*)‐1‐phenyl‐hexa‐1,5‐dien‐3,4‐diol (Lombardo et al., 2001). The absolute configuration of the diol motif remained undetermined.

ARP DM104 was assigned a molecular formula of C_12_H_15_NO_3_ based on the observed ion [M + H]^+^ at 222.1135 (calcd. For C_12_H_16_NO_3_^+^ = 222.1125, Δm = 4.5 ppm), indicating six degrees of unsaturation. Its UV–vis (DAD) spectrum differed from that of the compounds described above. Analysis of its ^1^H and HSQC NMR spectra revealed two aromatic and two olefinic hydrogens, two oxygenated methine groups, two aliphatic methylenes and finally an aliphatic methyl in vinylic position. Two spin systems were identified in the COSY spectrum, one belonging to an aromatic ring and the second corresponding to an aliphatic substituent of the former (Figure 5). The COSY correlation of the two aromatic hydrogens was weak, indicating a small *J* coupling typical of their *meta* positioning. The observed key HMBC correlations together with the molecular formula and the ^13^C NMR spectrum allowed stablishing the connectivity of the compound and secured the ring closure between C5 and C9 via and ether bridge (Figure 5). Since the two olefinic hydrogens are essentially isochronous, the configuration of this double bond could not be established from NOE or *J* coupling data. Nevertheless, it is safe to assume an *E* configuration for such double bond as for the argimycins described above, assuming a common biosynthesis route for that moiety. The absence of NOE correlation between H10 and methyl H13 further supports this proposal. The relative configuration of the two vicinal oxymethines could not be established due to the lack of measurable coupling constants (because signal broadening and overlap with the water signal). However, it can be assumed to be the same *syn* relative configuration (9R*, 10R*) indicated before for ARP DE45, again based on biosynthetic arguments. The absolute configuration was not determined but should match that of ARP DE45.

**FIGURE 6:**
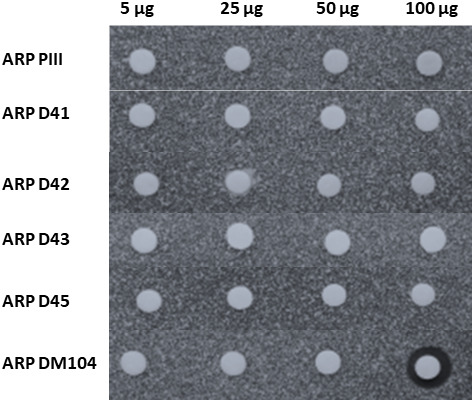
Bioassay of novel argimycin compounds. Bioassay against *Micrococcus luteus*. ARP, argimycins P.

The antibiotic activity of all novel ARP compounds was tested against *M. luteus*. It is worth mentioning that ARP D43 sample contains a mixture of three compounds, being the major one ARP D43. In this assay, we also included ARP PIII (also named as nigrifactin) as control, since it had shown the highest antibiotic activity among all ARP tested before (Ye et al., 2017). Among all compounds assayed, ARP DM104 was the only one that showed a clear antibiotic activity at the highest amount of compound tested (100 μg), while the same amount of ARP PIII did not show antibiotic activity (Figure 6).

## DISCUSSION

The ARP family of compounds includes piperidine and pyridine‐containing alkaloids, some of which contain one heterocycle attached to a polyene chain, while others also contain a five‐membered fused ring with a shorter polyene chain. To date, 13 ARP compounds have been identified and chemically characterized from cultures of *S. argillaceus* WT and knockout mutants in *arp* genes (Ye et al., 2017, 2018). In this work, we have been able to expand the number and scaffold diversity of ARP family through combinatorial biosynthesis. To do that, we took advantage of the fact that the ARP and CPK biosynthesis pathways have similar initial steps that lead to structurally similar aminated polyketide chains, diverting their pathways afterwards (Figure 2). Therefore, by expressing *cpkD* or *cpkDE* genes involved in later steps of CPK biosynthesis into *S. argillaceus* WT novel ARP derivatives were generated, either containing a quinolizidine backbone (ARP D41, ARP D42 and ARP D43) or a pyridine ring (ARP DE45) (Figure 5). In addition, by expressing *cpkD* into *S. argillaceus* MARPHI mutant strain the novel compound ARP DM104 with a pyranopyridine scaffold was produced. Although the precise steps for the biosynthesis of these novel ARP remain unclear, the heterologous expression of *cpkD* and *cpkDE* genes in *S. argillaceus* knockout mutants have revealed some hints about the involvement of some Arp proteins in the biosynthesis of these novel compounds (Figure 2C). Thus, ARP DE45 would derive from an early dehydrated ARP biosynthesis intermediate synthesized by the ArpP PKS and the ArpN ϖ‐transaminase; and the quinolizidine and pyranopyridine ARP compounds would do from an ArpDHI product. Also, it is discarded a role of cyclases ArpHI/HII in the cyclization events leading to the formation of quinolizidine and pyranopyridine scaffolds, pointing out that processes leading to formation of their second ring would occur spontaneously and not by a dedicated Arp enzyme.

Based on the structure of these novel ARP derivatives, it is proposed that CpkD would introduce an epoxide group at the C9‐C10 double bond either on an ArpDHI product (ARP D41‐ARP 43 and ARP DM104) or on an earlier intermediate (ARP DE45). CpkE might be responsible for the subsequent hydrolysation of the epoxide on that generated intermediate to produce ARP DE45 (Figure 2C). CpkE has been proposed to be a putative isomerase whose action would facilitate formation of the heterocycle in CPK (Gomez‐Escribano et al., 2012). However, a BlastP search against the Protein Data Bank (PDB) reveals similarity with epoxide hydrolases, activity that fits better to its proposed role in the formation of ARP DE45. Moreover, individual expression of *cpkE* does not lead to any new compound, suggesting that its coding enzyme would act after CpkD, what would support a coordinated activity between CpkD and CpkE. Since the other two flavin‐dependent epoxidases/dehydrogenases (CpkH and ScF) encoded by the *cpk* BGC do not modify the ARP biosynthetic pathway when expressed alone in *S. argillaceus*, it is proposed that in CPK biosynthesis, CpkD would be involved in epoxidation of the C9‐C10 double bond in the corresponding CPK intermediate, which would occur before epoxidation of C7‐C8 double bond by either CpkH or ScF.

Four of the novel ARP derivatives are bicyclic showing unprecedented scaffolds never found before in the ARP family of compounds. Thus, ARP D41 to ARP D43 show a bicyclic quinolizidine scaffold with an unusual iminium ion, in which the six‐membered fused ring is formed by an unnatural cyclization between C10 and the nitrogen atom in the heterocycle, instead of the natural cyclization between C9 and C5 that leads to the formation of five‐membered fused ring of bicyclic ARPs. All these three quinolizidine ARPs share a hydroxy group at C7 but differ at the heterocycle. Quinolizidines constitute a major class of alkaloid compounds that are mainly produced by plants, in which the biosynthesis pathway starts with the amino acid lysine (Bunsupa et al., 2012; Michael, 2008). Noticeable, in this work we have set up an alternative pathway for the biosynthesis of quinolizidine compounds using as precursor an aminated polyketide chain (Figure 2C), by creating a hybrid pathway combining genes from two different but related BGCs each of which unable to direct the biosynthesis of a quinolizidine (Gomez‐Escribano et al., 2012; Ye et al., 2017). Another novel scaffold in ARPs corresponds to ARP DM104, which contains a pyranopyridine backbone. Most probably this scaffold would derive from a biosynthesis intermediate accumulated by mutant MARPHI and produced as result of ArpDHI activity, which after being modified by CpkD would suffer dehydration of hydroxy groups at C5 and C9 (Figure 2C).

We have previously reported that most ARP do not show antibiotic activity, except ARP PIII that shows a very weak antibiotic activity against *M. luteus* (Ye et al., 2017). Now we report that ARP DM104, apart from bearing an unprecedented scaffold in ARP compounds, shows improved activity against *M. luteus*. Thus, combinatorial biosynthesis applied to polyketide alkaloids has proven as a successful strategy for the generation of structural diversity and enhanced bioactivity in ARP, paving the way for the exploitation of an unexplored source of new compounds.

## AUTHOR CONTRIBUTIONS

**Suhui Ye:** Conceptualization (equal); investigation (equal); writing – original draft (equal). **Giovanni Ballin:** Investigation (equal). **Ignacio Pérez‐Victoria:** Investigation (equal). **Alfredo Férnandez Braña:** Investigation (supporting). **Jesús Martín:** Investigation (supporting). **Fernando Reyes:** Investigation (supporting). **Jose A Salas:** Conceptualization (equal); funding acquisition (equal). **Carmen Méndez:** Conceptualization (equal); formal analysis (lead); funding acquisition (lead); supervision (lead); writing – original draft (lead); writing – review and editing (lead).

## FUNDING INFORMATION

This work was supported by grants to CM from the Spanish Ministry of Economy and Competitiveness, MINECO (Grants BIO2014‐56752‐R and PIM2010EEI‐00752) and by the grant “Apoyo a Grupos de Excelencia”, Principado de Asturias‐FEDER (FC‐15‐GRUPIN14‐014).

## CONFLICT OF INTEREST

The authors declare no competing financial interest.

## Supporting information


Appendix S1
