# Supplementary material for: Combinatorial biosynthesis yields novel hybrid argimycin P alkaloids with diverse scaffolds in Streptomyces argillaceus
Source: Microb Biotechnol. 2022 Nov 8;15(12):2905–16. doi: 10.1111/1751-7915.14167 (PMC9733639; doi:10.1111/1751-7915.14167)
Supplement: Supplementary file 1 — Appendix S1 [file MBT2-15-2905-s001.doc]

**SUPPORTING INFORMATION**

**Combinatorial biosynthesis yields novel hybrid argimycin P alkaloids with diverse scaffolds in *Streptomyces argillaceus***

**Suhui Ye^†‡^, Giovanni Ballin****^†^, Ignacio Pérez-Victoria^#^, Alfredo F. Braña^†^, Jesús Martín^#^, Fernando Reyes^#^, José A. Salas^†‡^ and Carmen Méndez^†‡^***

**^†^Departamento de Biología Funcional e Instituto Universitario de Oncología del Principado de Asturias (I.U.O.P.A), Universidad de Oviedo, Oviedo, Spain**

**^‡^Instituto de Investigación Sanitaria de Asturias (ISPA), Oviedo, Spain**

**^#^Fundación MEDINA, Centro de Excelencia en Investigación de Medicamentos Innovadores en Andalucía, Armilla, Granada, Spain.**

*** Correspondence: Carmen Méndez (**[**cmendezf@uniovi.es**](mailto:cmendezf@uniovi.es)**). Phone number: +34 985103558**

**Running title**: Hybrid argimycins P by combinatorial biosynthesis

**Plasmid constructs for expressing *cpk* genes**

Several plasmids were generated by amplifying *cpk* genes from *S. coelicolor* using oligonuecleotides from Table S1, and subcloned in either pSETEc or pSETETc downstream of the erythromycin resistance promoter, as follows:

pSETEcScF: *ScF* was amplified using oligonucleotides ScFA/ScFB, subcloned into pCR-Blunt and released as a BglII-EcoRI fragment to be subcloned into pSETEc digested with BamHI and EcoRI.

pSETEcCpkH: *cpkH* was amplified using oligonucleotides CpkHA/CpkHB, subcloned into pCR-Blunt and released as a BamHI-EcoRI fragment to be subcloned into the same sites of pSETEc.

pSETEcCpkD/pSETETcCpkD: *cpkD* was amplified using oligonucleotides CpkDA/CpkDB, subcloned into pCR-Blunt and released as a BamHI-EcoRI fragment to be subcloned into the same sites of pSETEc and pSETETc.

pSETEcCpkDE/pSETETcCpkDE: *cpkD* and *cpkE* were amplified as a sole fragment using oligonucleotides CpkDA/CpkEB, subcloned into pCR-Blunt, generating the construct pCRBluntCpkDE, and released as an EcoRI fragment to be subcloned in the right orientation into the same site of pSETEc and pSETETc.

pSETEcCpkE: to generate this plasmid, pCRBluntCpkDE was digested with PvuI and EcoRI, and the PvuI-EcoRI fragment containing *cpkE* was purified and fill ends with Klenow. Then, the resulting blunt-ended fragment was subcloned in the right orientation into the EcoRV site of pSETEc.

**Table S1.** Oligonucleotide sequences used in this work

| **PRIMER** | **SEQUENCE 5’-3’** |
| --- | --- |
| **ScFA**  **ScFB**  **CpkHA**  **CpkHB**  **CpkDA**  **CpkDB**  **CpkEB** | AGATCTCTCTCTTGTTCTCACGG  GAATTCCTGTGGCTACGGCTAC  GGATCCCCGCAATTCCTGGTCAGT  GAATTCTGCGGGGAGGGTCAG  GGATCCCTTGTTGTTCCCACTCC  GAATTCCGAAGACCAGGCCGT  GAATTCGTTCTCTGTGCCTGTGG |

**Table S2.** NMR data for ARP D41 (CD_3_OD, 24 °C, 500 MHz/125 MHz)

| Position | δ_H_ (ppm), mult. (*J* in Hz) | δ_C_ (ppm), type |  |
| --- | --- | --- | --- |
| 2 | 4.59, app t (7.6) | 47.9, CH_2_ |
| 3 | 2.76, app ddd (12.3, 7.8, 1.5) | 21.8, CH_2_ |
| 4 | 6.80, ddd (9.9, 4.6, 4.6) | 135.7, CH |
| 5 | 7.09, dt (9.9, 1.5) | 116.8, CH |
| 6 |  | 136.0, C |
| 7 |  | 151.4, C |
| 8 | 7.79, d (8.8) | 129.6, CH |
| 9 | 7.74, d (8.8) | 125.2, CH |
| 10 |  | 144.6, C |
| 11 | 6.83, dq (15.4, 1.7) | 121.6, CH |
| 12 | 6.62, dq (15.4, 6.7) | 140.2, CH |
| 13 | 2.07, dd (6.7, 1.7) | 17.8, CH_3_ |

**Table S3.** NMR data for ARP D42 (CD_3_OD, 24 °C, 500 MHz/125 MHz)

| Position | δ_H_ (ppm), mult. (*J* in Hz) | δ_C_ (ppm), type |  |
| --- | --- | --- | --- |
| 2 | 9.23, d (7.1) | 132.2, CH |
| 3 | 8.04, app t (7.1) | 123.8, CH |
| 4 | 8.28, app t (8.0) | 134.2, CH |
| 5 | 8.78, d (8.6) | 122.6, CH |
| 6 |  | 136.2, C |
| 7 |  | 152.1, C |
| 8 | 7.64, d (8.5) | 117.2, CH |
| 9 | 7.93, d (8.5) | 123.9, CH |
| 10 |  | 136.2, C |
| 11 | 6.99, br d (15.5) | 121.4, CH |
| 12 | 6.66, dq (15.4, 6.7) | 138.9, CH |
| 13 | 2.13, dd (6.7, 1.6) | 17.7, CH_3_ |

**Table S4.** NMR data for ARP D43 (CD_3_OD, 24 °C, 500 MHz/125 MHz)

| Position | δ_H_ (ppm), mult. (*J* in Hz) | δ_C_ (ppm), type |  |
| --- | --- | --- | --- |
| 2 | 4.63, ddd (14.2, 9.0, 5.1)  4.52, app dt (14.2, 5.5) | 48.8, CH_2_ |
| 3 | 2.29, dddd (14.2, 8.7, 5.2, 3.1)  2.19, dddd (14.2, 6.2, 5.5, 4.6) | 28.6, CH_2_ |
| 4 | 4.39, dddd (6.2, 4.6, 4.6, 3.2) | 59.2, CH |
| 5 | 3.30, dd (19.1, 4.6)  3.20, br dd (19.1, 4.2) | 32.1, CH_2_ |
| 6 |  | 142.6, C |
| 7 |  | 154.0, C |
| 8 | 7.74, d (8.8) | 127.5, CH |
| 9 | 7.77, d (8.8) | 124.0, CH |
| 10 |  | 144.5, C |
| 11 | 6.82, dq (15.5, 1.7) | 121.8, CH |
| 12 | 6.59, dq (15.4, 6.7) | 139.3, CH |
| 13 | 2.06, dd (6.7, 1.6) | 17.7, CH_3_ |

**Table S5.** NMR data for ARP D44 (CD_3_OD, 24 °C, 500 MHz/125 MHz)

| Position | δ_H_ (ppm), mult. (*J* in Hz) | δ_C_ (ppm), type |  |
| --- | --- | --- | --- |
| 2 | 3.66, app t (5.7) | 43.3, CH_2_ |
| 3 | 2.00, app quint. (6.1) | 19.1, CH_2_ |
| 4 | 2.66, app tt (6.5, 1.5) | 19.1, CH_2_ |
| 5 |  | 129.4, C |
| 6 |  | 187.2, C |
| 7 | 3.03, m^a^ | 28.4, CH_2_ |
| 8 | 3.02, m^a^ | 29.1, CH |
| 9 |  | 169.4, C |
| 10 | 6.87, br d (15.7) | 120.3, CH |
| 11 | 6.70, dd (15.7, 4.8) | 150.1, CH |
| 12 | 4.53, dq (6.7, 4.8) | 67.1, CH |
| 13 | 1.33, d (6.7) | 21.6, CH_3_ |

Chemical shifts of quaternary carbons were retrieved from the HMBC spectrum

**Table S6.** NMR data for ARP DE45 (CD_3_OD, 24 °C, 500 MHz/125 MHz)

| Position | δ_H_ (ppm), mult. (*J* in Hz) | δ_C_ (ppm), type |  |
| --- | --- | --- | --- |
| 2 | 8.66, br d (5.9) | 141.5, CH |
| 3 | 7.82, app t (6.7) | 124.4, CH |
| 4 | 8.45, app td (8.0, 1.2) | 145.2, CH |
| 5 | 8.17, br d (8.2) | 123.8, CH |
| 6 |  | 151.0 |
| 7 | 6.95, dd (16.0, 1.7) | 121.6, CH |
| 8 | 7.24, dd (16.0, 4.5) | 144.3, CH |
| 9 | 4.32, app td (5.0, 1.8) | 74.3, CH |
| 10 | 4.10, app br t (6.0) | 75.4, CH |
| 11 | 5.63, ddq (15.3, 6.9, 1.7) | 130.2, CH |
| 12 | 5.80, dqd (15.3, 6.6, 1.0) | 128.4, CH |
| 13 | 1.75, dd (6.6, 1.5) | 16.6, CH_3_ |

**Table S7.** NMR data for ARP DM104 (CD_3_OD, 24 °C, 500 MHz/125 MHz)

| Position | δ_H_ (ppm), mult.^a^ (*J* in Hz) | δ_C_ (ppm), type |  |
| --- | --- | --- | --- |
| 2 | 7.88, br s | 137.7, CH |
| 3 |  | 159.7, C |
| 4 | 7.01, br s | 111.1, CH |
| 5 |  | 142.9, C |
| 6 |  | 139.9, C |
| 7 | 3.12, br m | 17.8, CH_2_ |
| 8 | 2.14, br m  2.02, br m | 20.4, CH_2_ |
| 9 | 4.23, br m | 65.7, CH |
| 10 | 4.91, m | 71.2, CH |
| 11 | 5.65, br s | 127.5, CH |
| 12 | 5.65, br s | 131.4, CH |
| 13 | 1.76, br d (2.8) | 16.4, CH_3_ |
| Note: severe signal broadening (due to possible paramagnetic contaminant) precluded determination of coupling constants in multiplets | | | |

**Figure S1.** Comparison of argimycins P (*arp*) and coelimycin P1 (*cpk*) biosynthesis gene clusters

**Figure S2.** UV-vis (DAD) spectrum of ARP D41

**Figure S3.** ESI-TOF HRMS spectrum of ARP D41


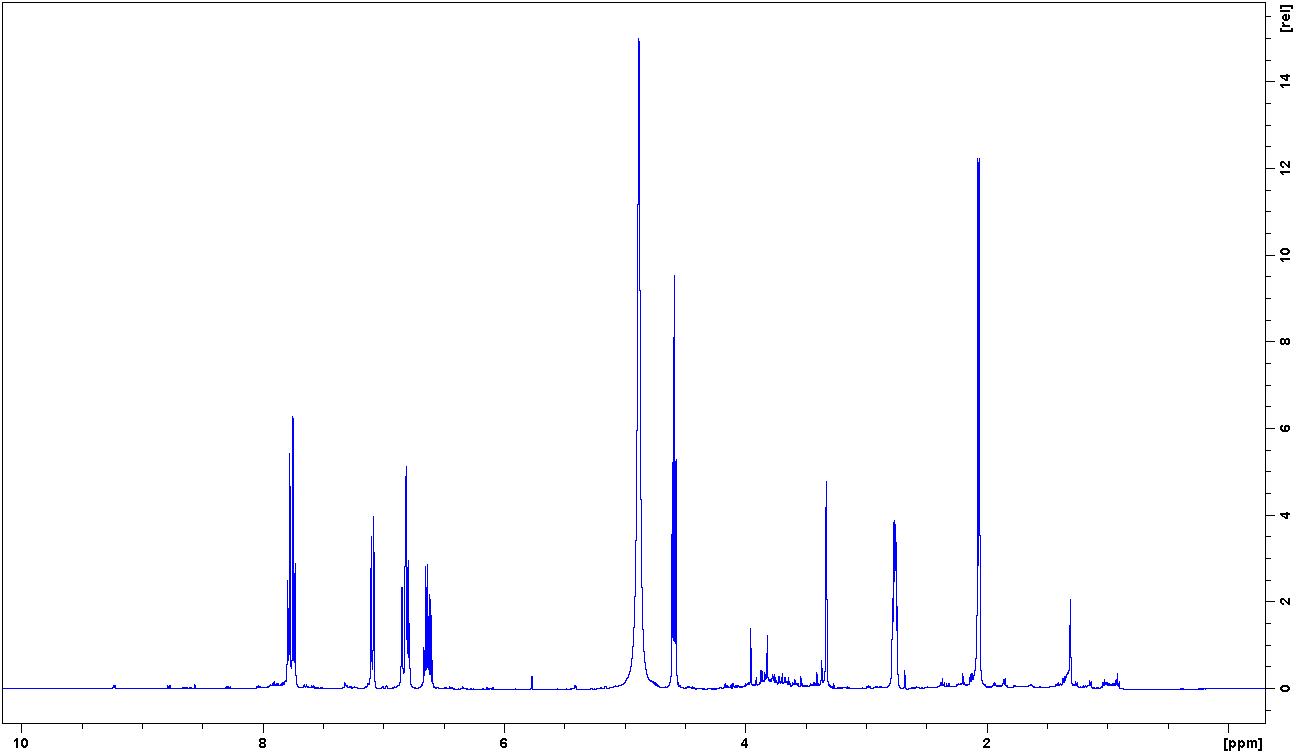


**Figure S4.** ^1^H spectrum of ARP D41 (CD_3_OD, 24 °C, 500 MHz)


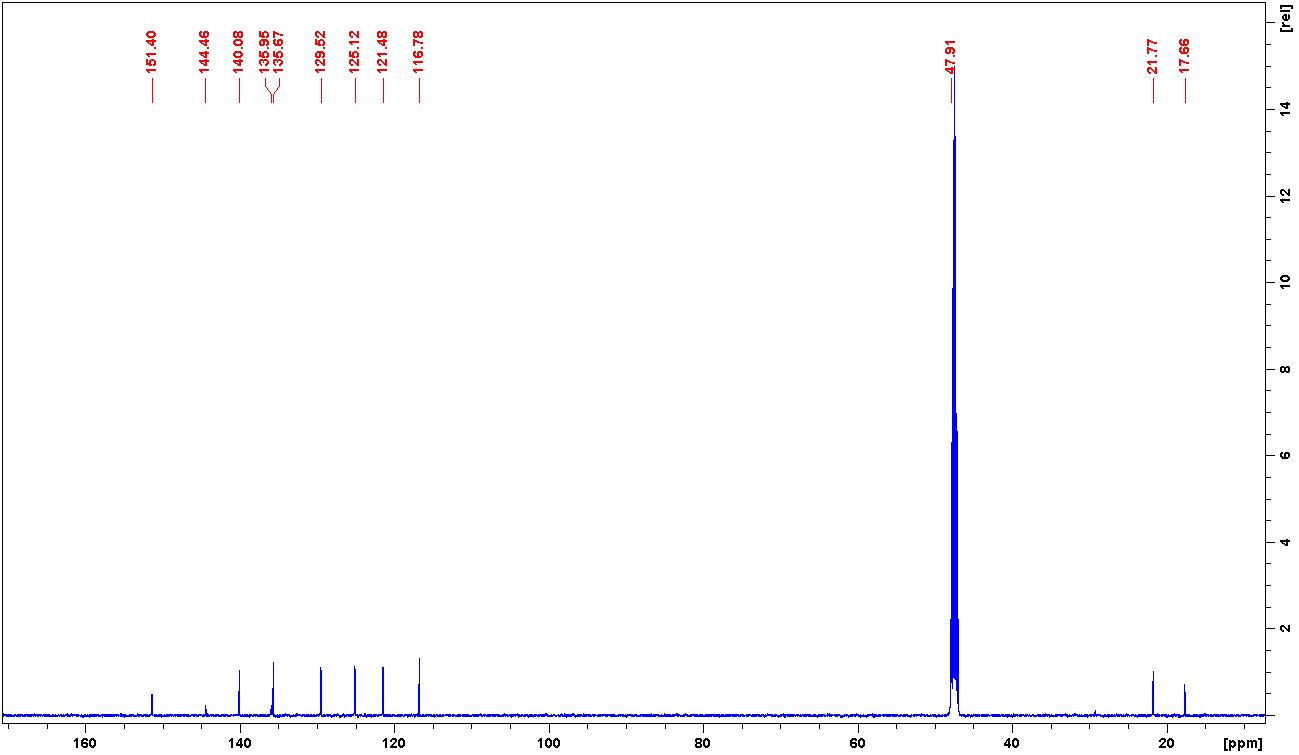


**Figure S5.** ^13^C spectrum of ARP D41 (CD_3_OD, 24 °C, 125 MHz)


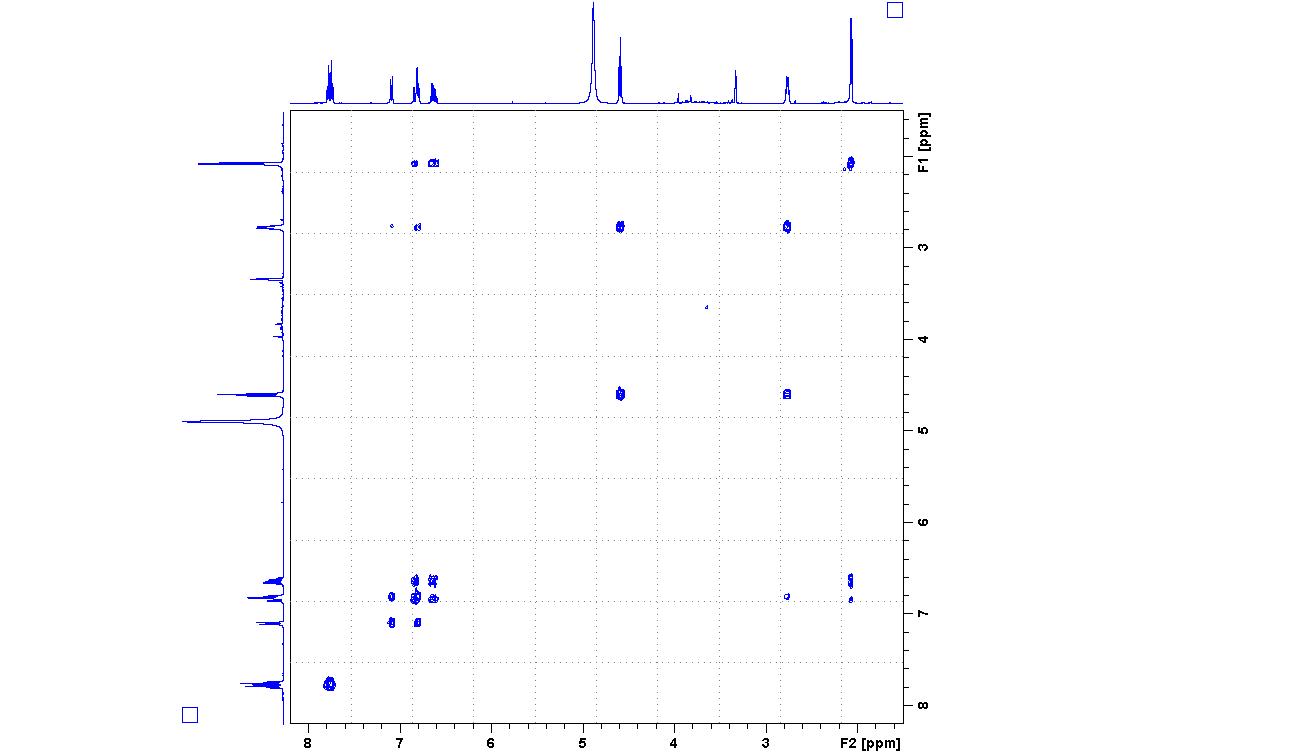


**Figure S6.** COSY spectrum of ARP D41


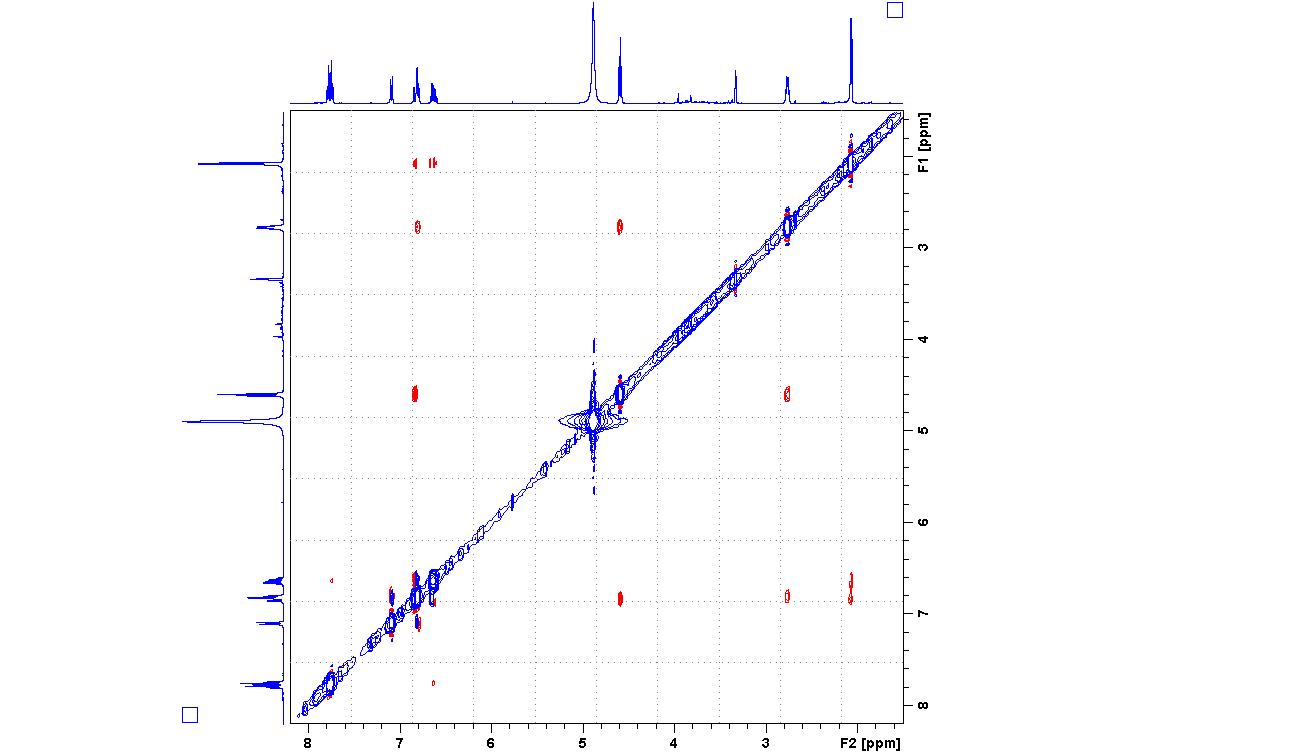


**Figure S7.** NOESY spectrum of ARP D41


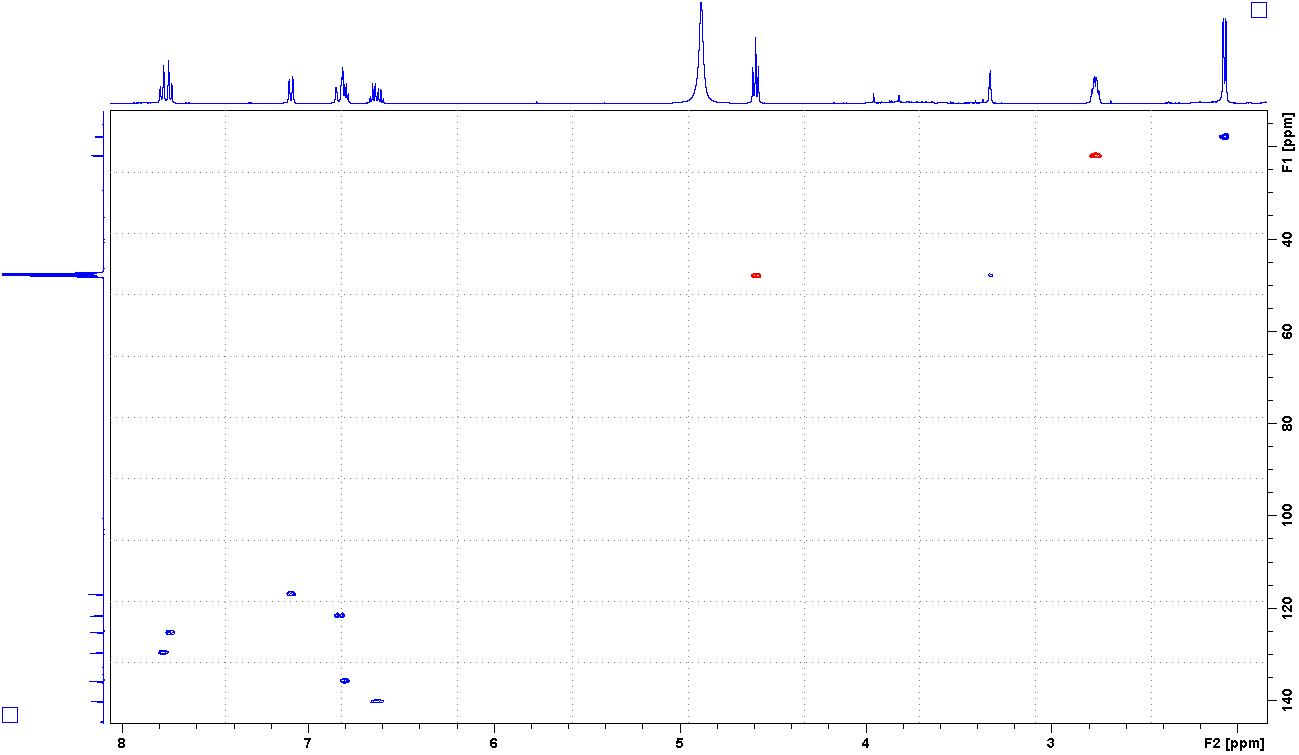


**Figure S8.** Edited HSQC spectrum of ARP D41


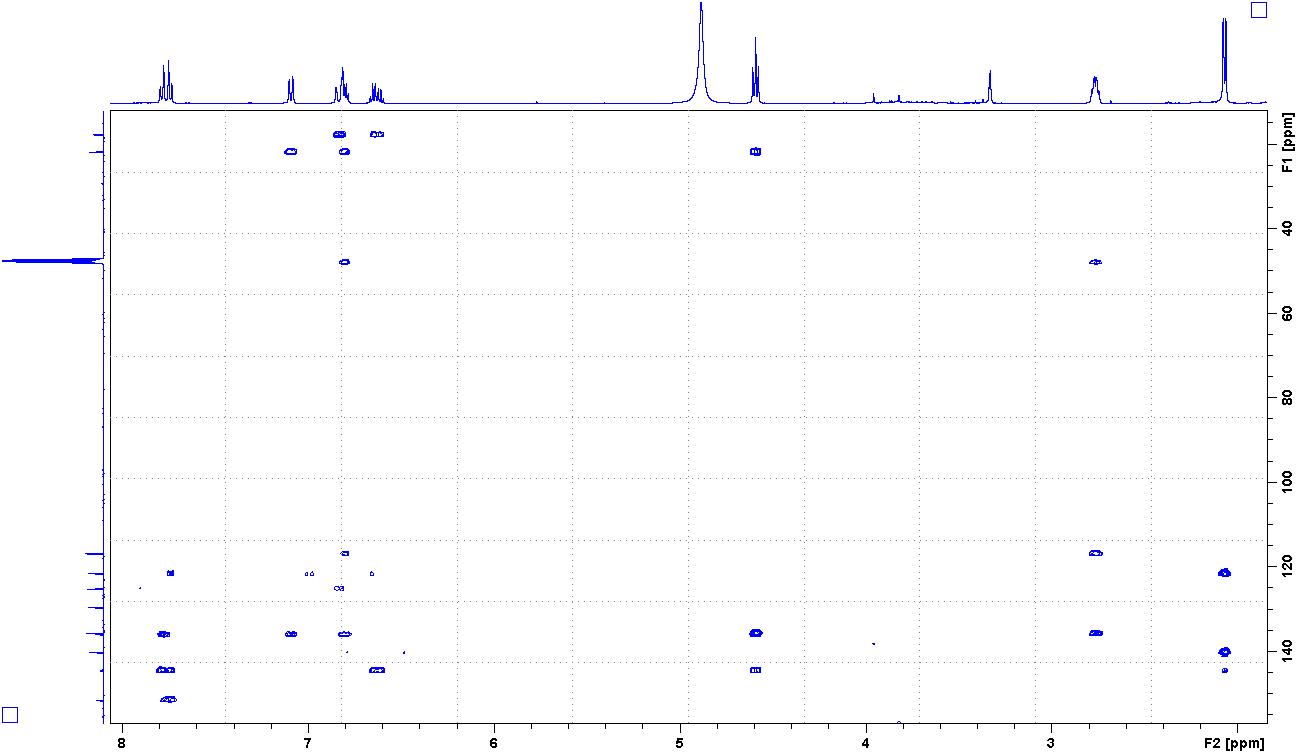


**Figure S9.** HMBC spectrum of ARP D41

**Figure S10.** UV-vis (DAD) spectrum of ARP D42

**Figure S11.** ESI-TOF HRMS spectrum of ARP D42


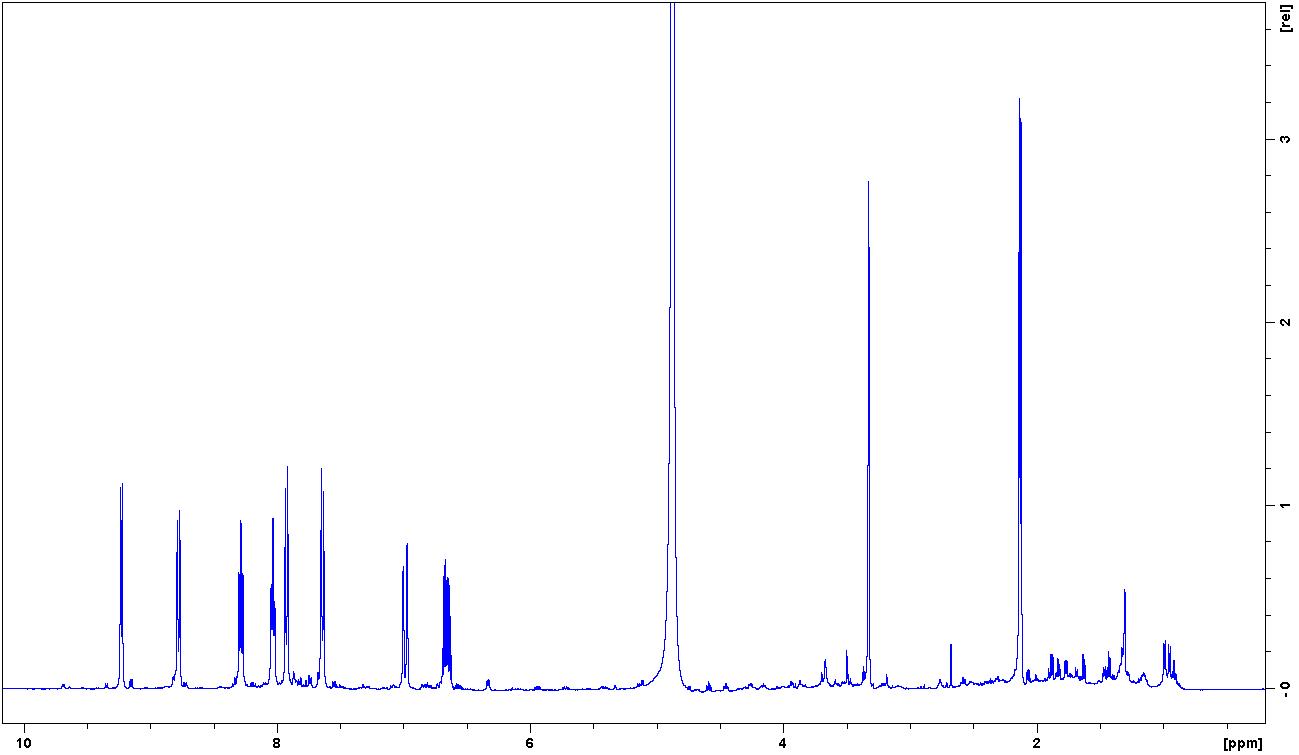


**Figure S12.** ^1^H spectrum of ARP D42 (CD_3_OD, 24 °C, 500 MHz)


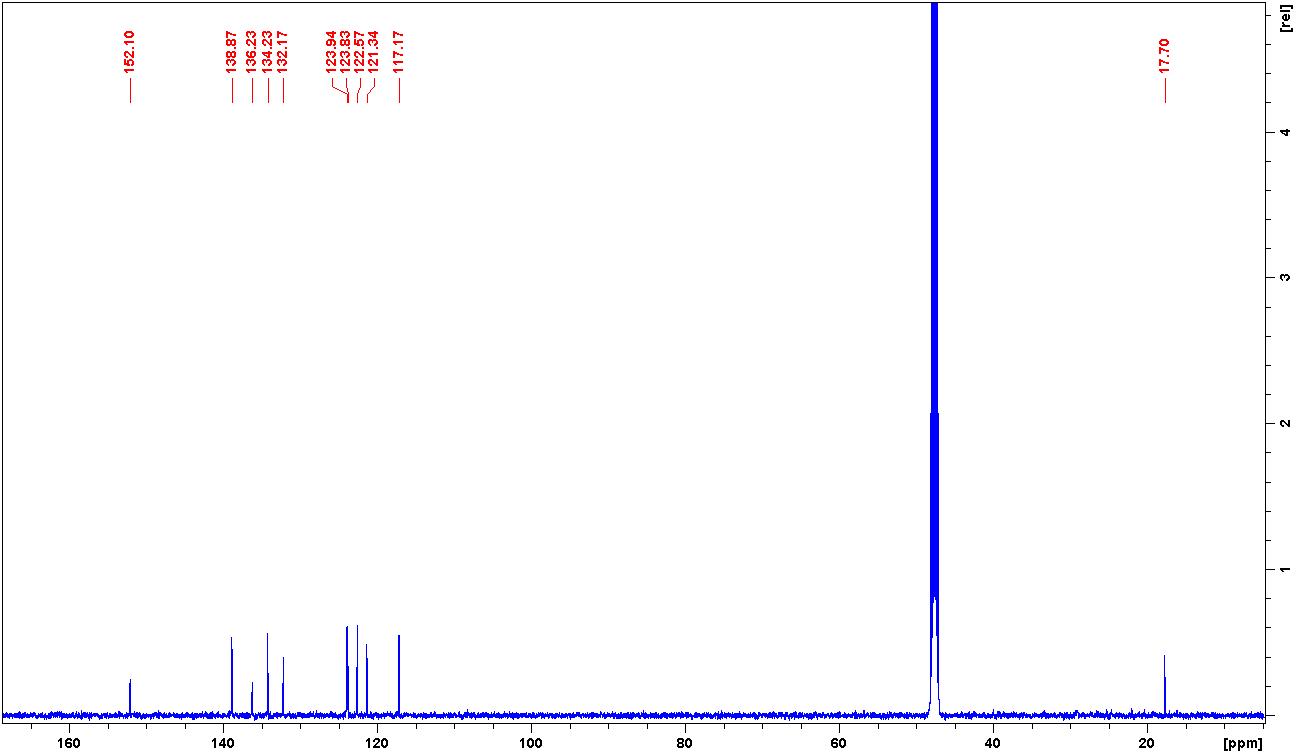


**Figure S13.** ^13^C spectrum of ARP D42 (CD_3_OD, 24 °C, 125 MHz)


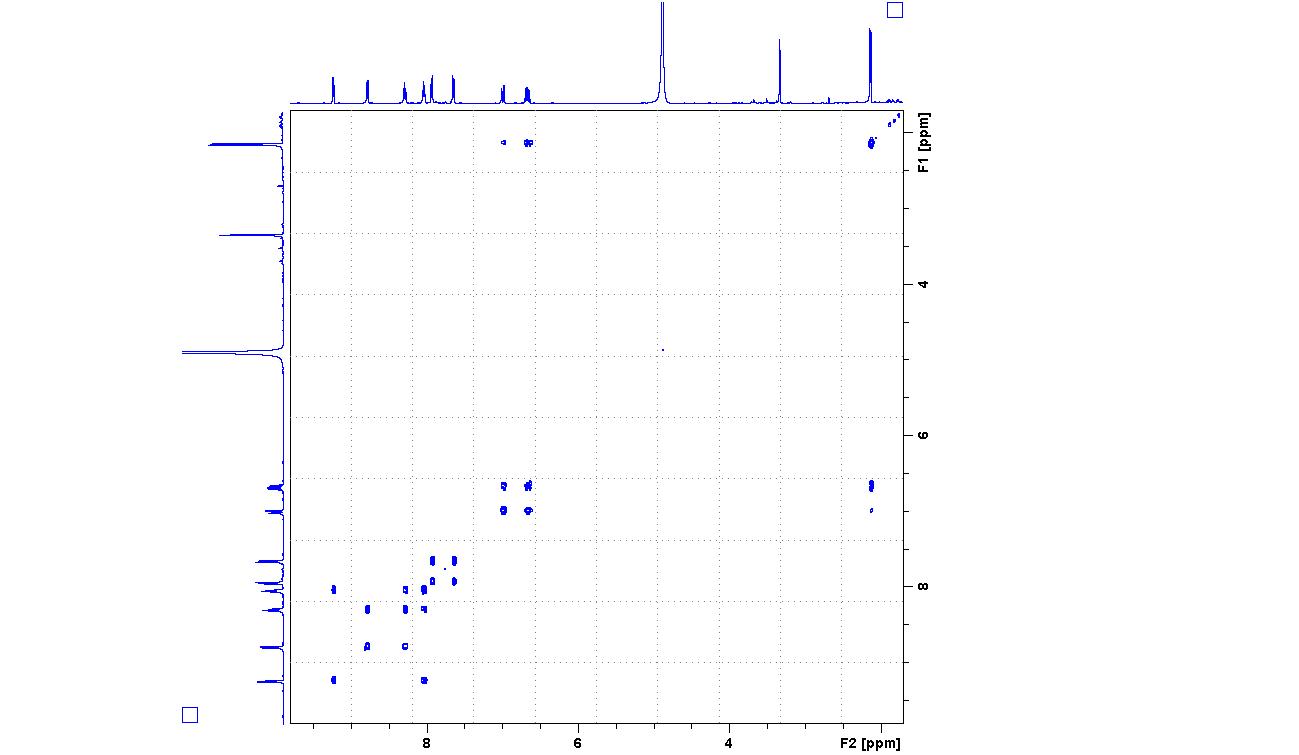


**Figure S14.** COSY spectrum of ARP D42


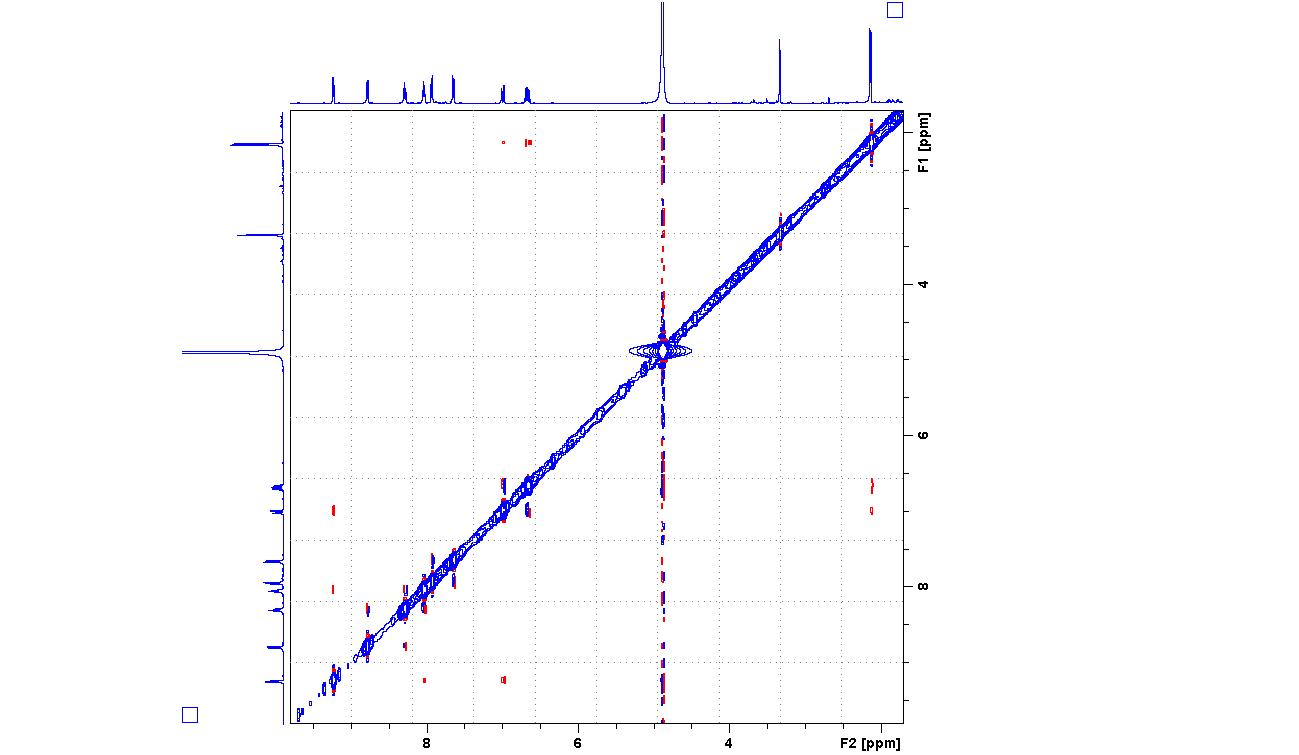


**Figure S15.** NOESY spectrum of ARP D42


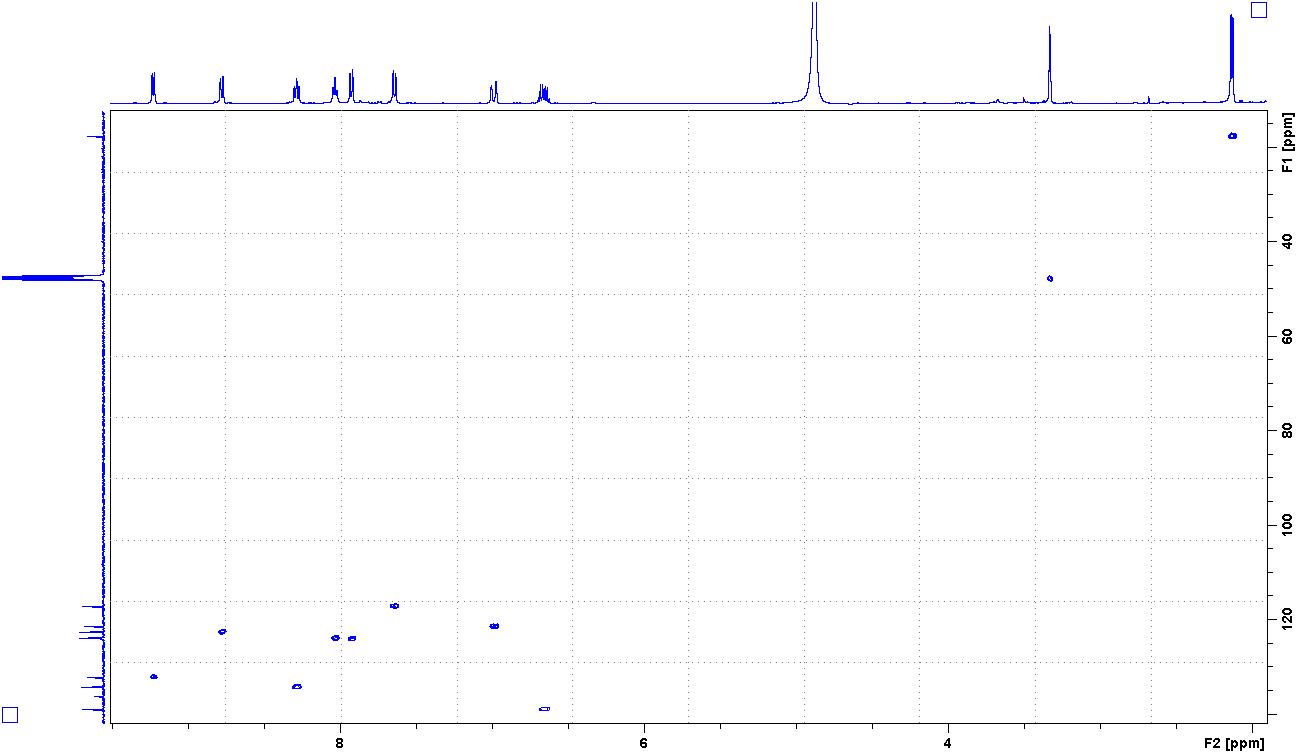


**Figure S16.** Edited HSQC spectrum of ARP D42


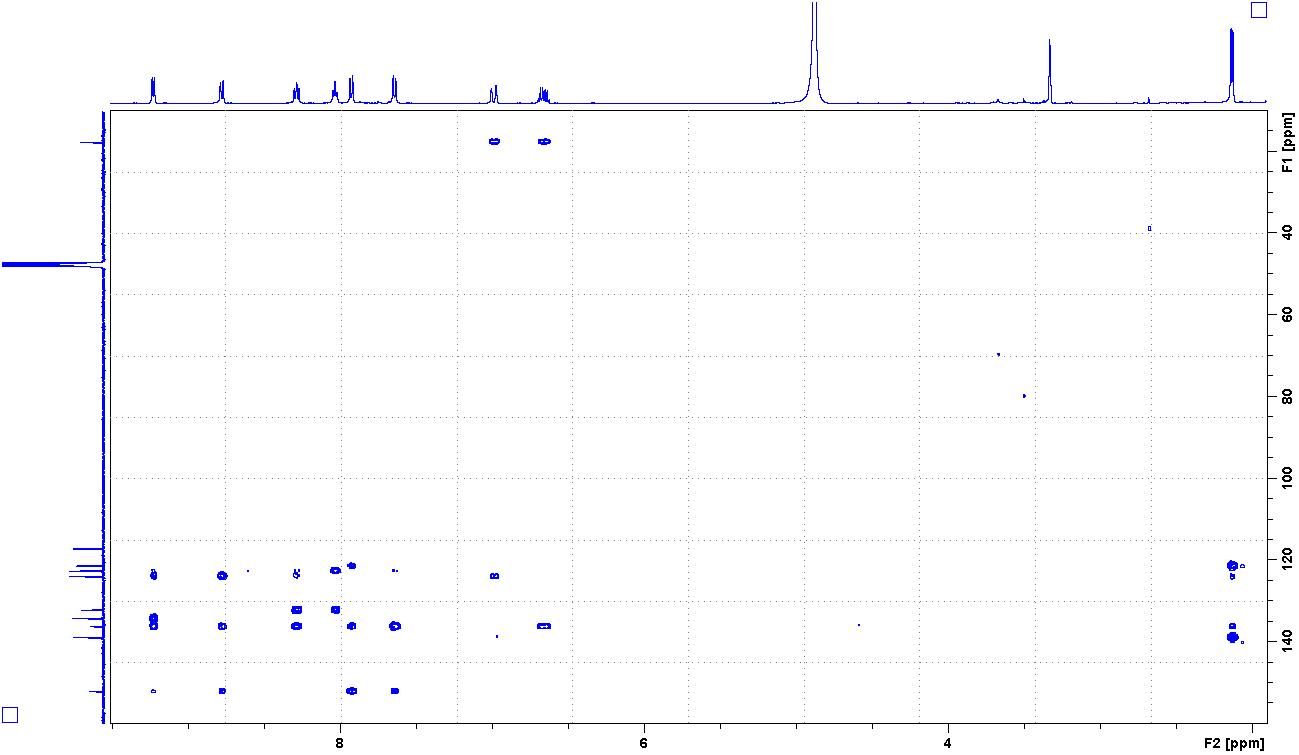


**Figure S17.** HMBC spectrum of ARP D42

**Figure S18.** UV-vis (DAD) spectrum of ARP D43

**Figure S19.** ESI-TOF HRMS spectrum of ARP D43


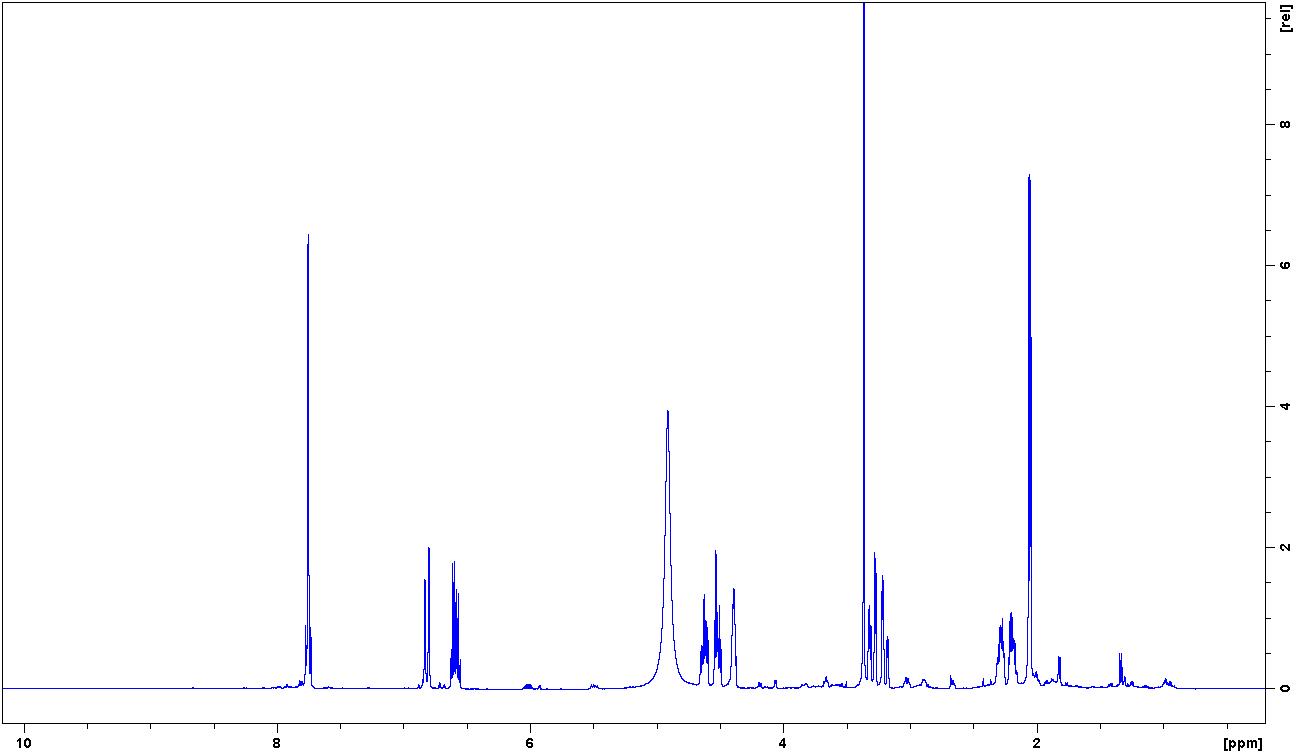


**Figure S20.** ^1^H spectrum of ARP D43 (CD_3_OD, 24 °C, 500 MHz)


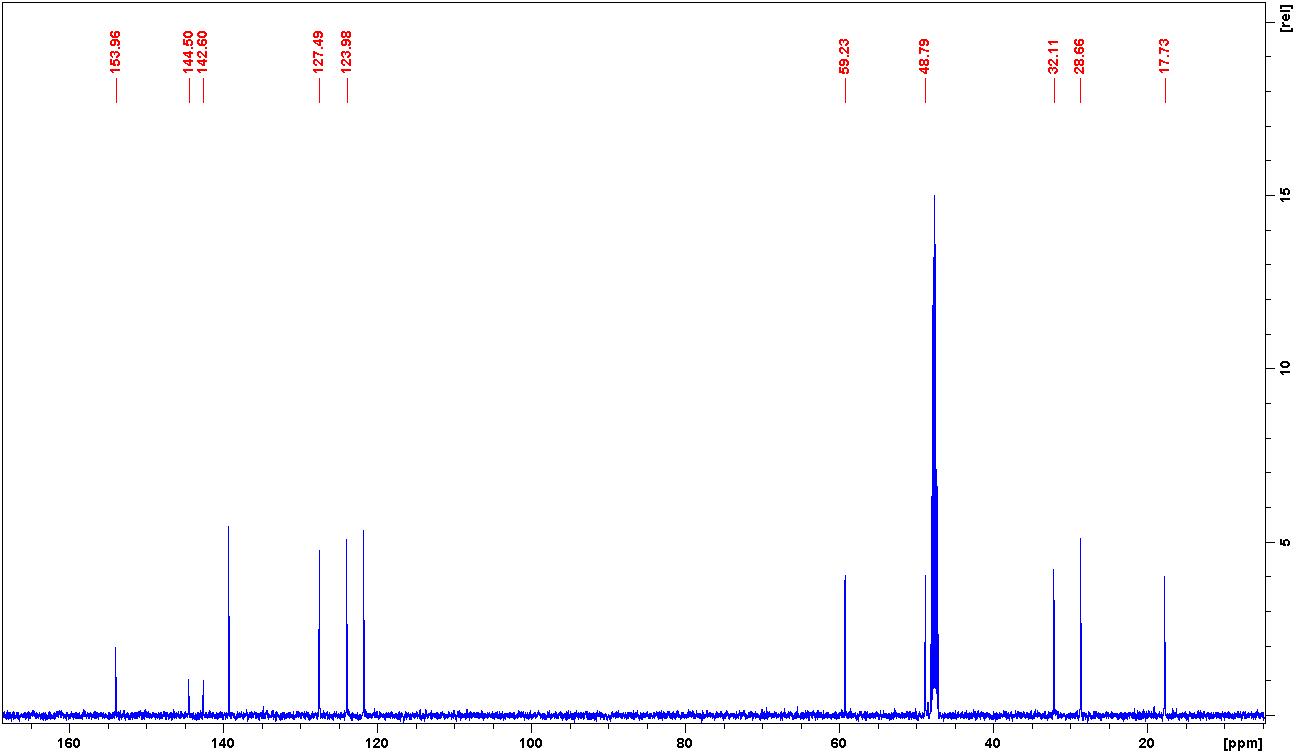


**Figure S21.** ^13^C spectrum of ARP D43 (CD_3_OD, 24 °C, 125 MHz)


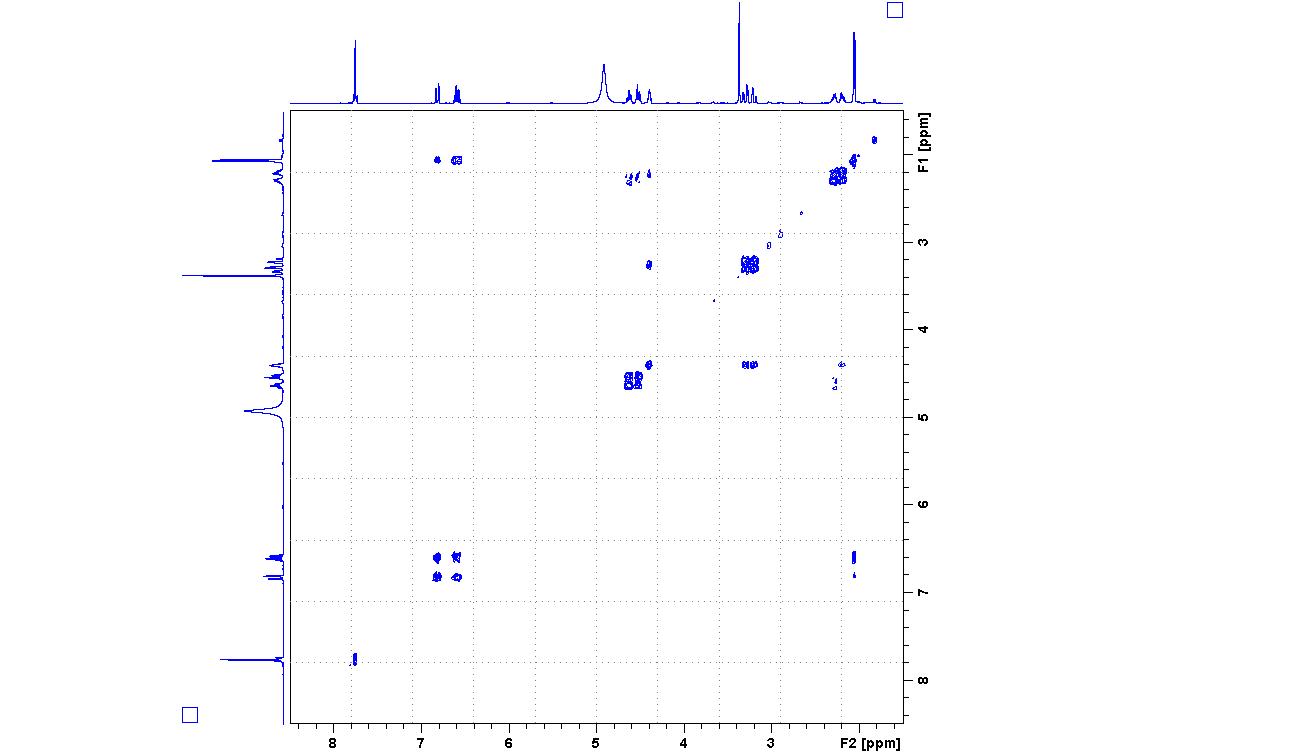


**Figure S22.** COSY spectrum of ARP D43


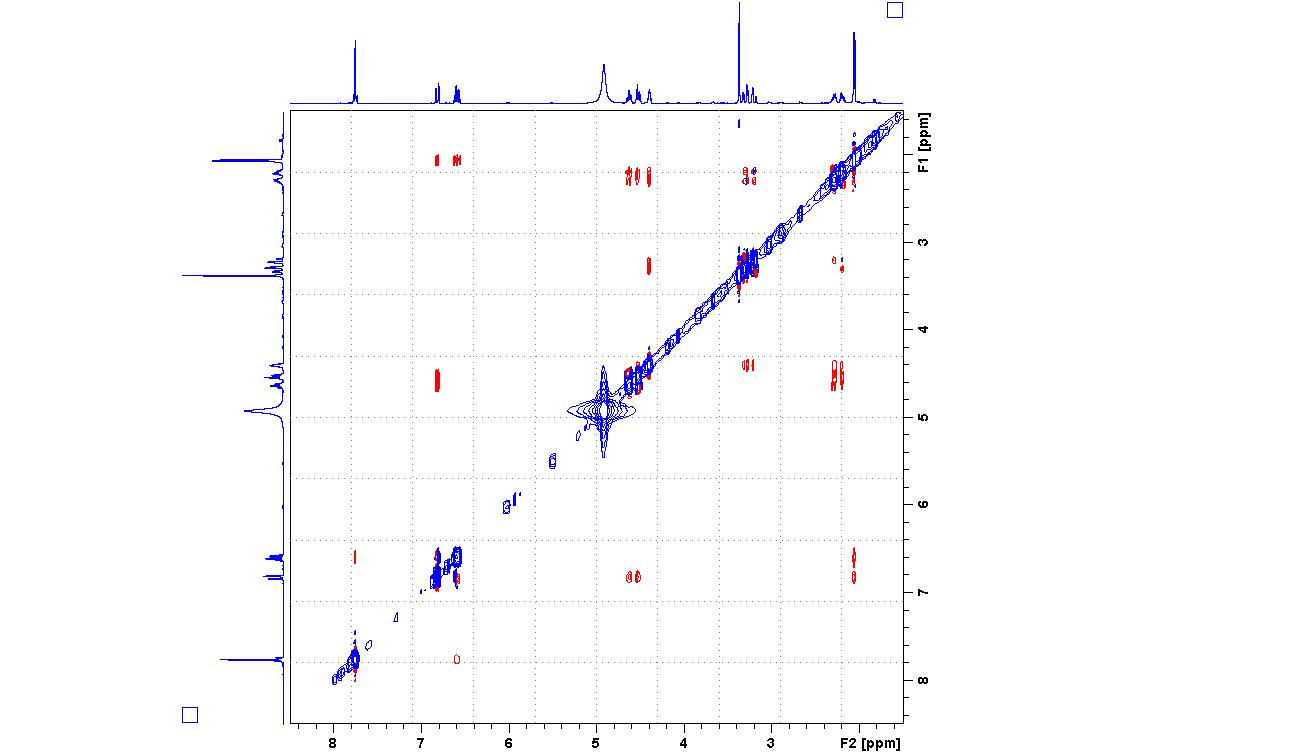


**Figure S23.** NOESY spectrum of ARP D43


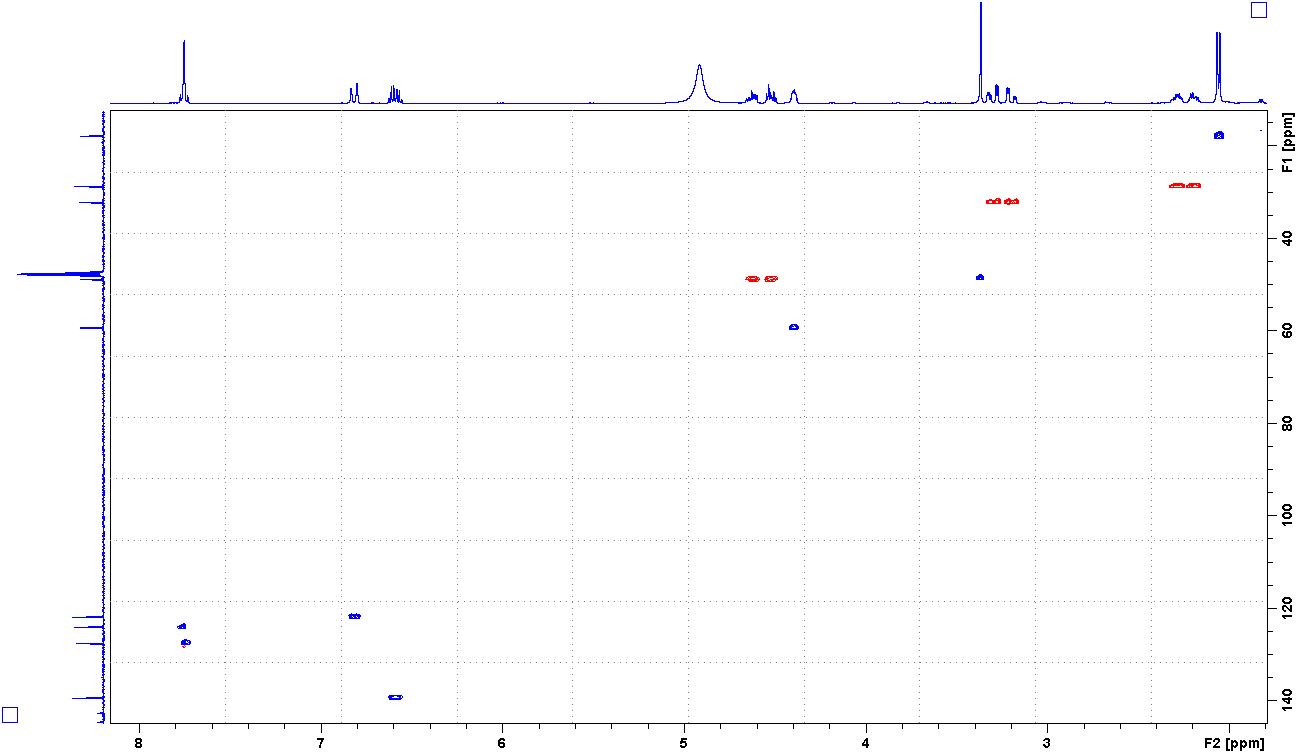


**Figure S24.** Edited HSQC spectrum of ARP D43


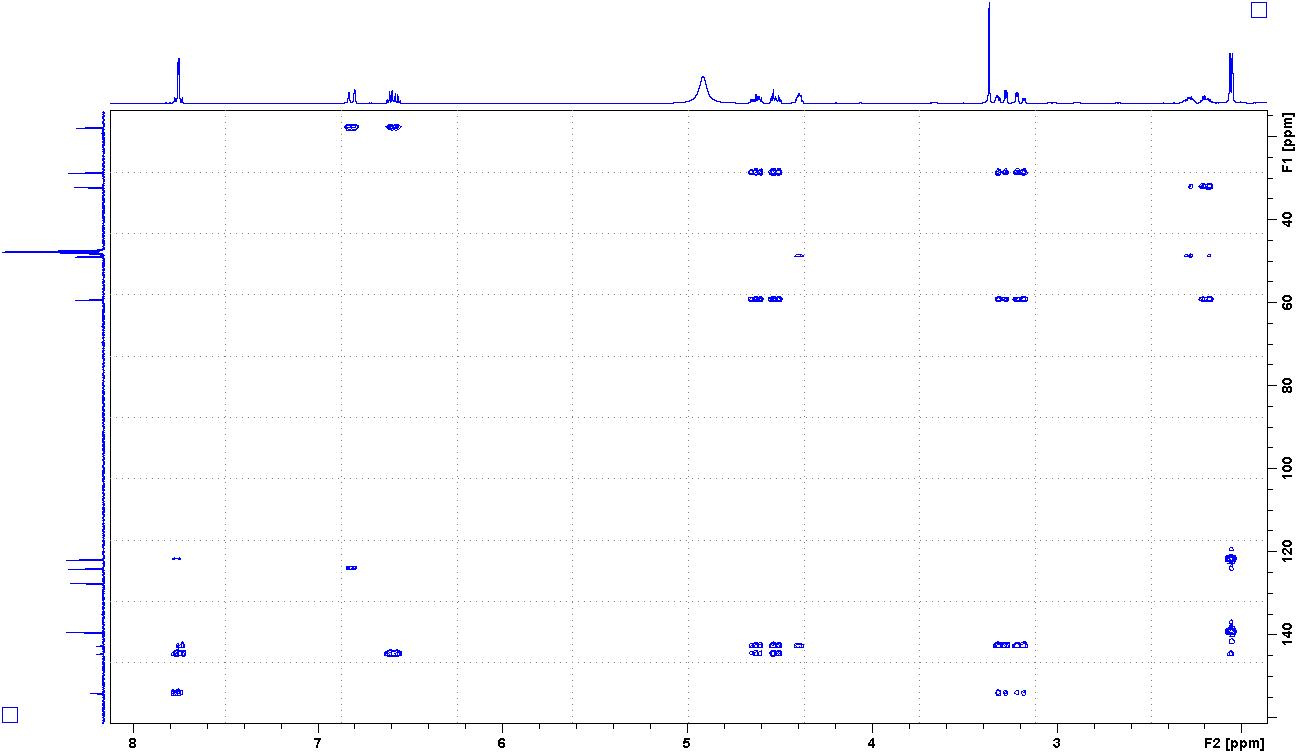


**Figure S25.** HMBC spectrum of ARP D43

**Figure S26.** UV-vis (DAD) spectrum of fraction containing ARP D44 as minor component

**Figure S27.** ESI-TOF HRMS spectrum of fraction containing ARP D44 as minor component


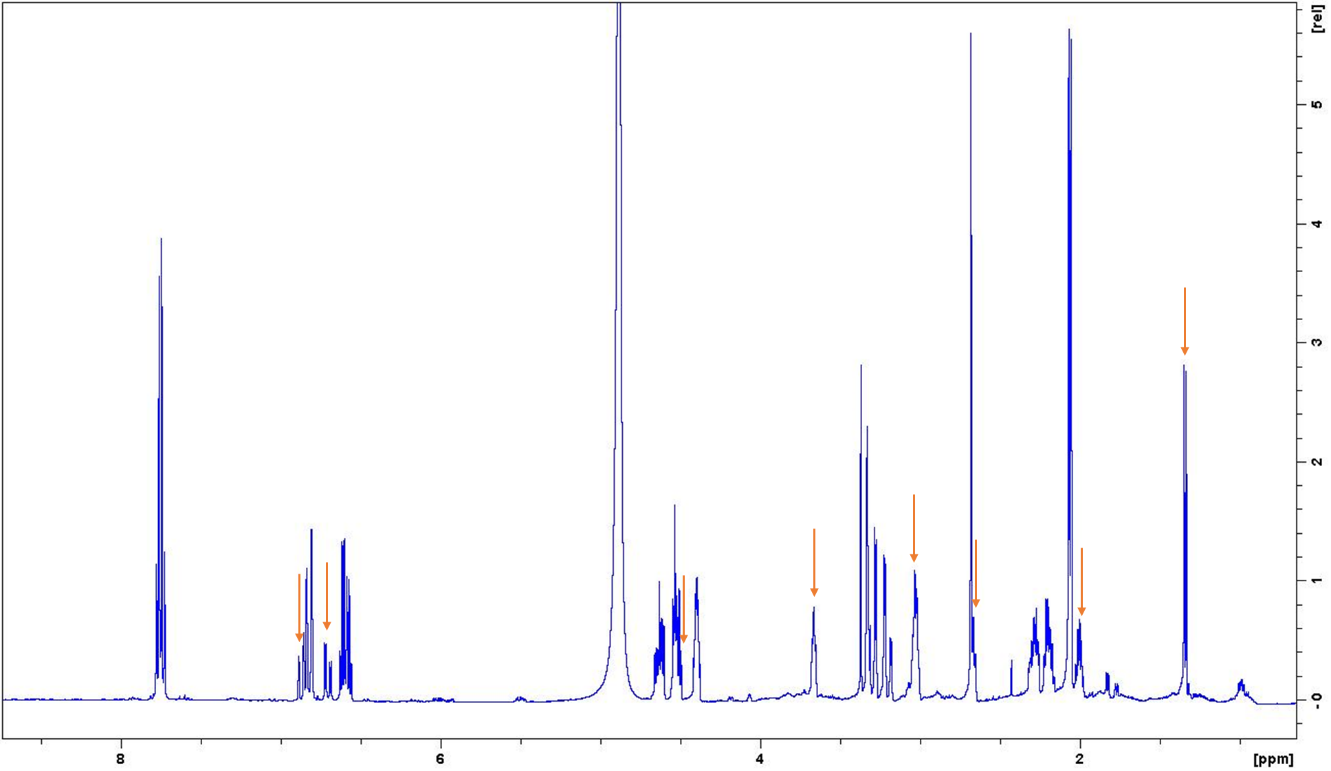


**Figure S28.** ^1^H spectrum of fraction containing ARP D44 as minor component (CD_3_OD, 500 MHz)


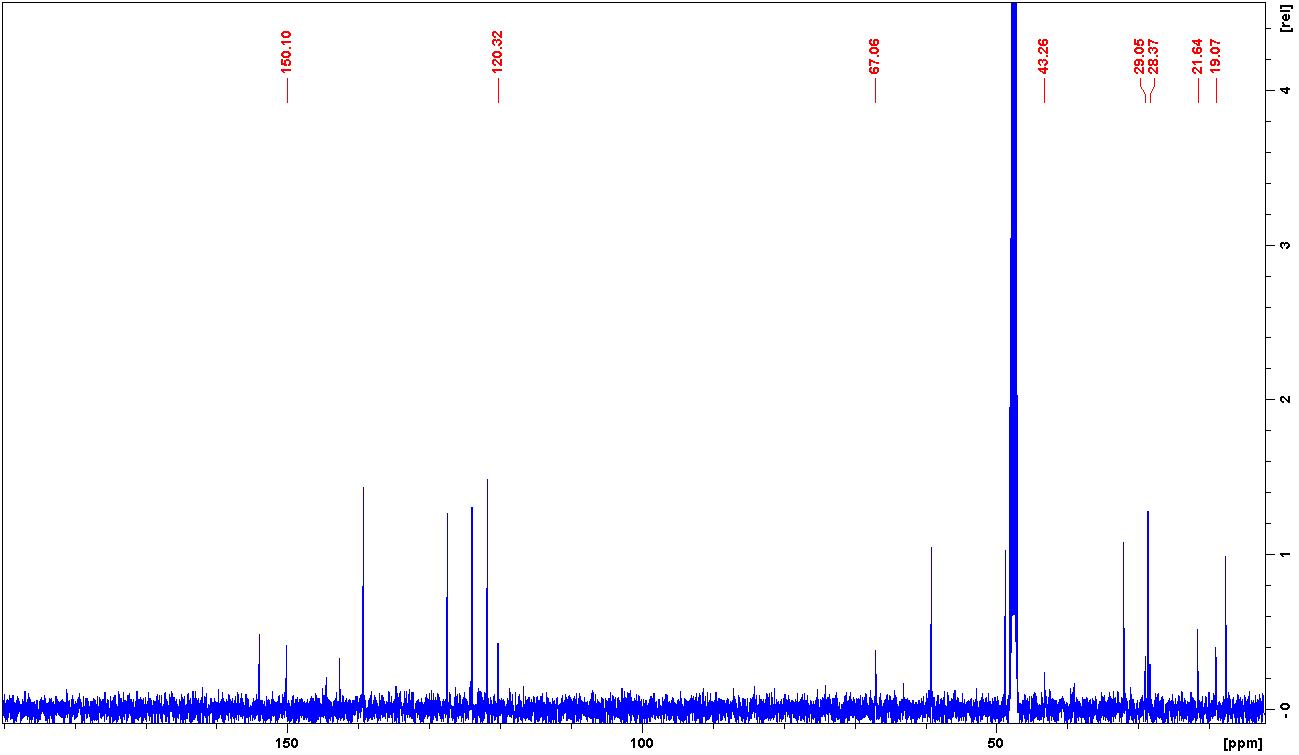


**Figure S29.** ^13^C spectrum of fraction containing ARP D44 as minor component (CD_3_OD, 500 MHz)


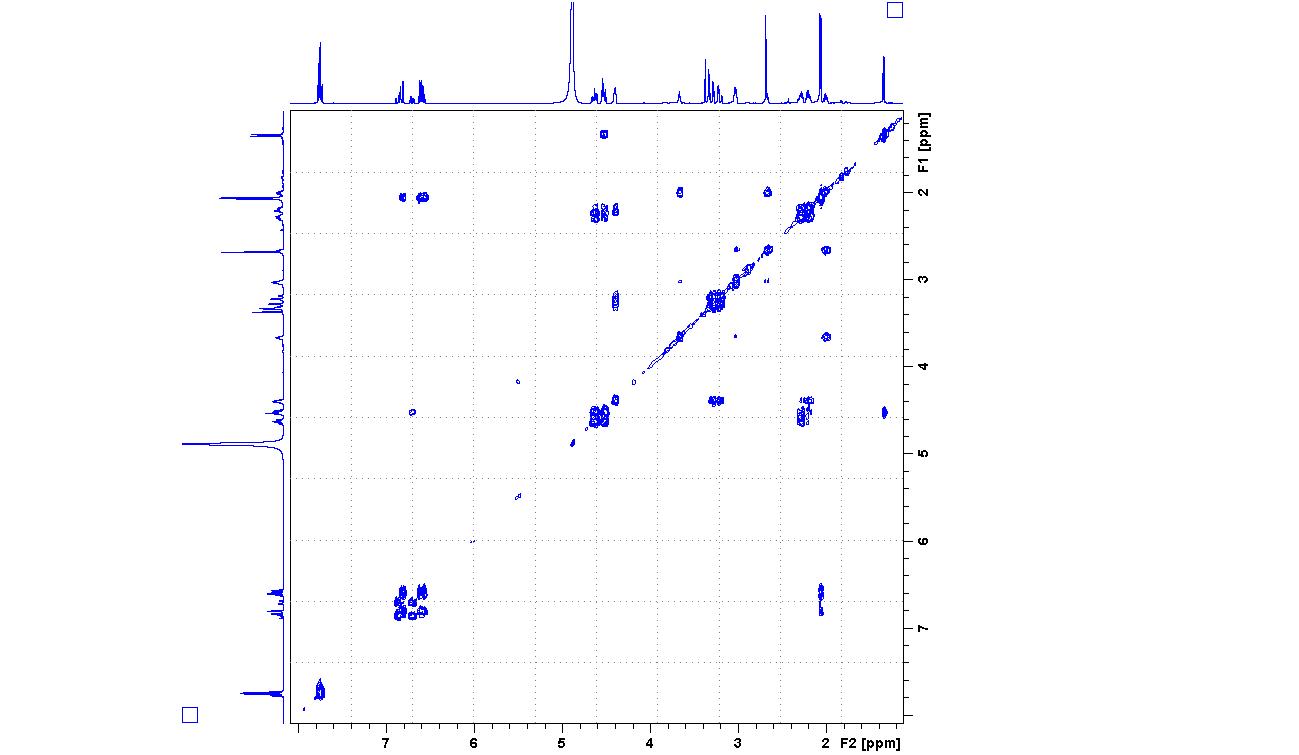


**Figure S30.** COSY spectrum of fraction containing ARP D44 as minor component


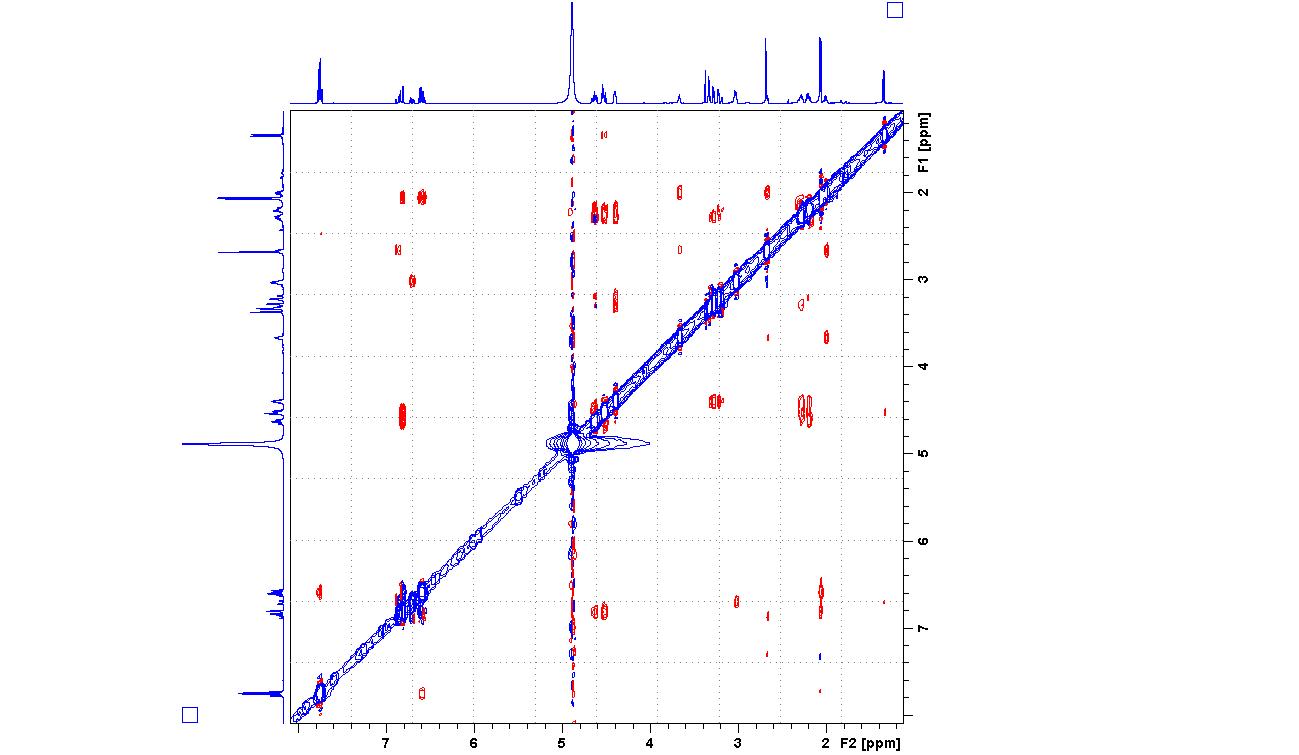


**Figure S31.** NOESY spectrum of fraction containing ARP D44 as minor component


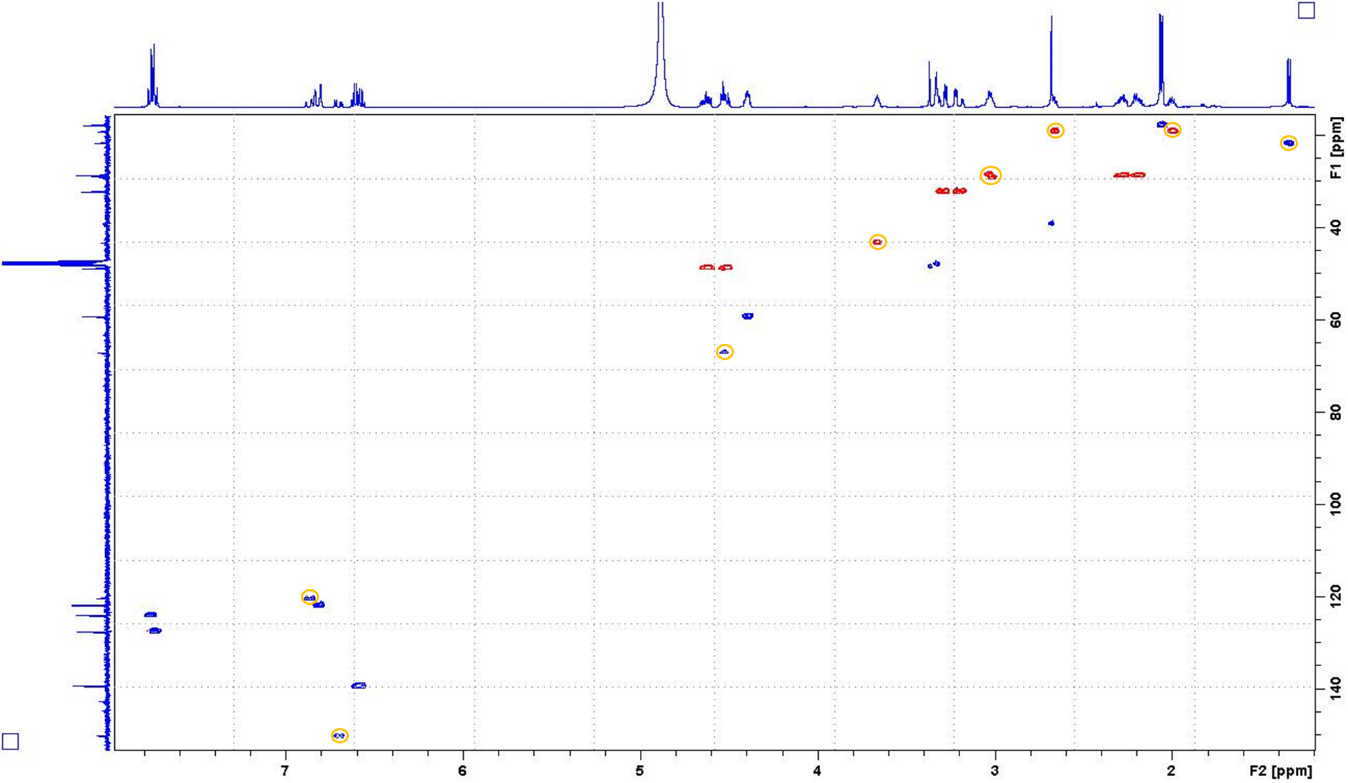


**Figure S32.** Edited HSQC spectrum of fraction containing ARP D44 as minor component


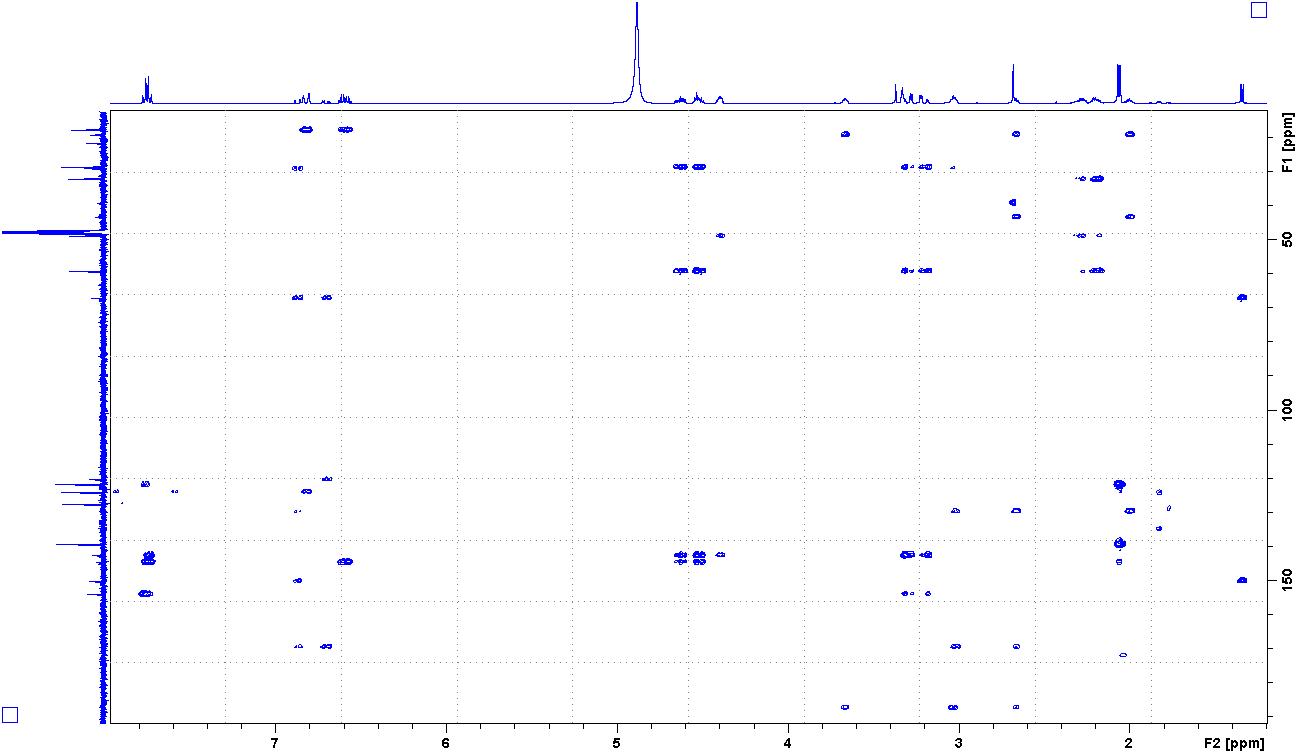


**Figure S33.** HMBC spectrum of fraction containing ARP D44 as minor component

**Figure S34.** UV-vis (DAD) spectrum of ARP DE45

**Figure S35.** ESI-TOF HRMS spectrum of ARP DE45


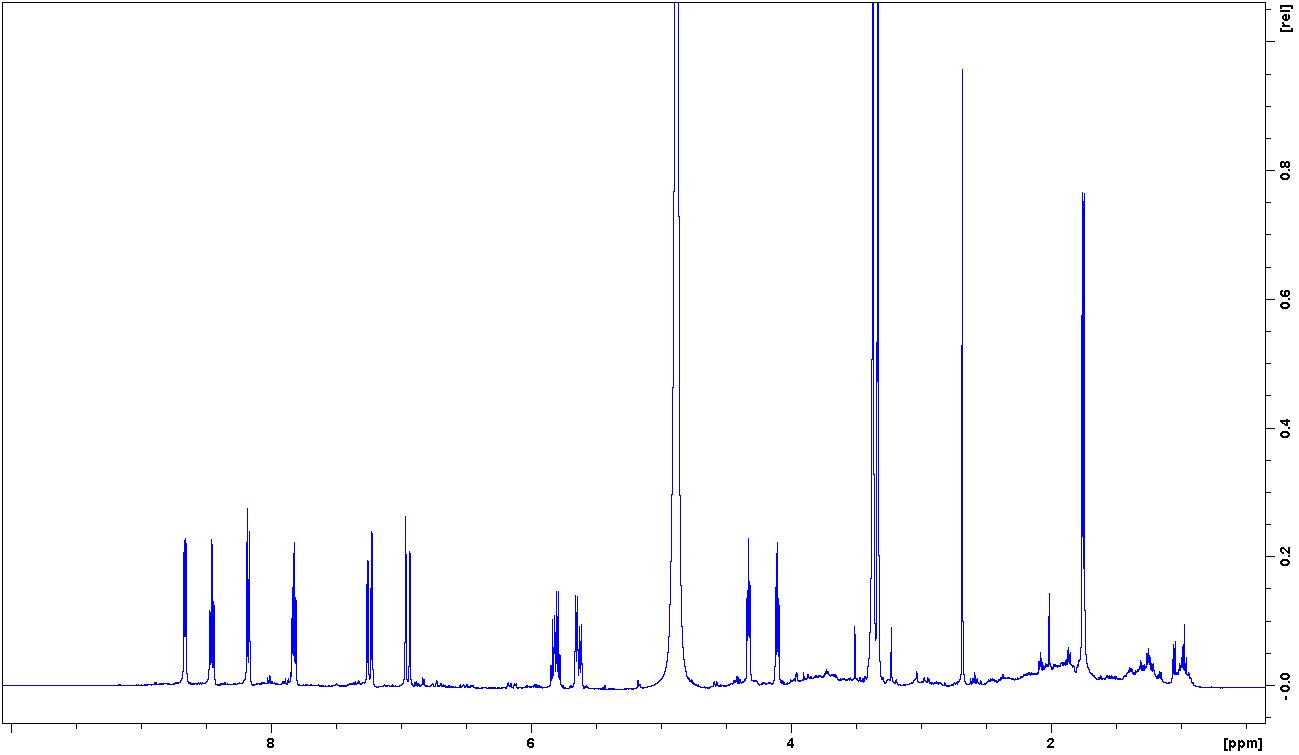


**Figure S36.** ^1^H spectrum of ARP DE45 (CD_3_OD, 24 °C, 500 MHz)


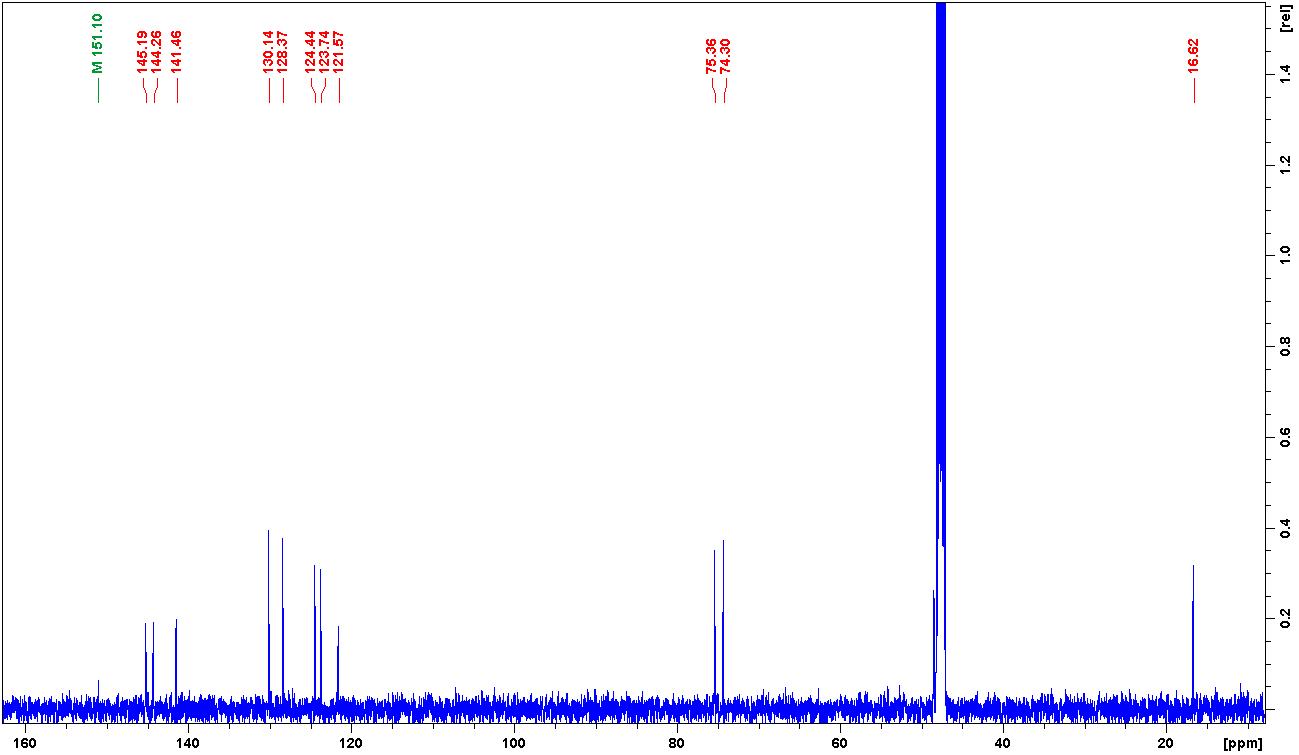


**Figure S37.** ^13^C spectrum of ARP DE45 (CD_3_OD, 24 °C, 125 MHz)


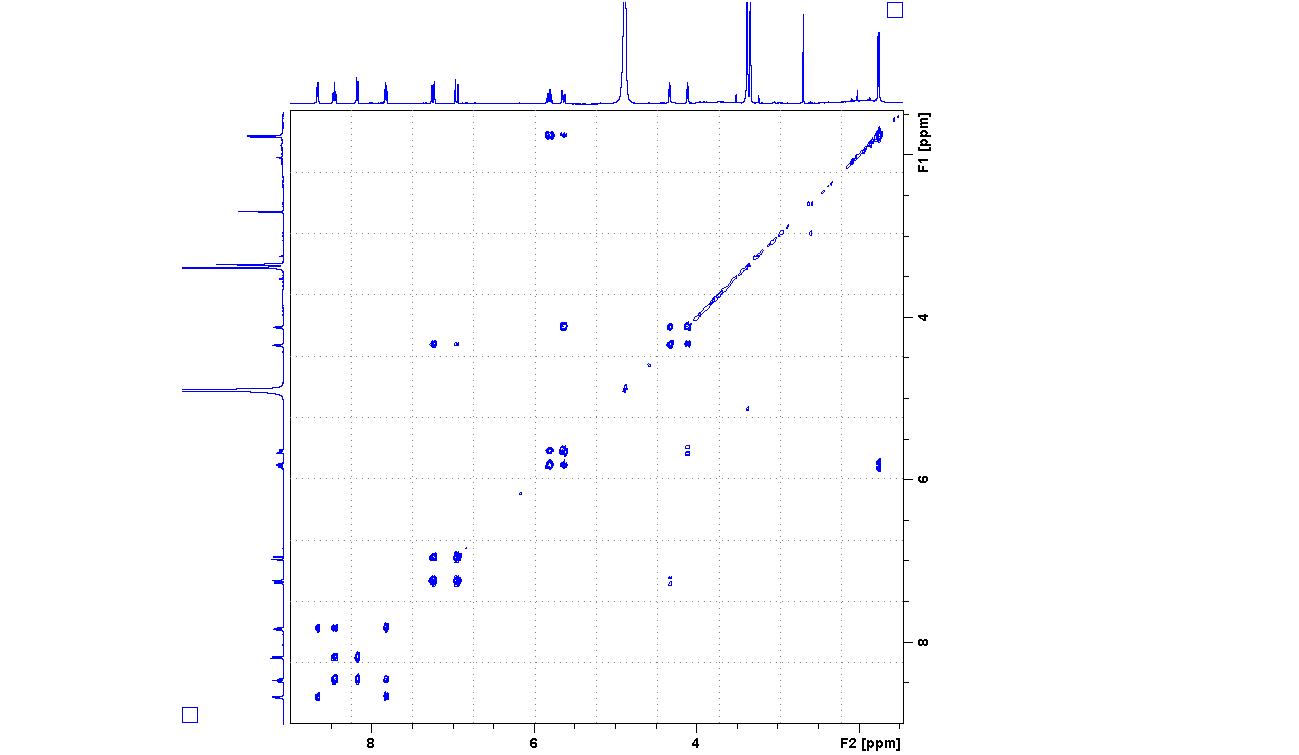


**Figure S38.** COSY spectrum of ARP DE45


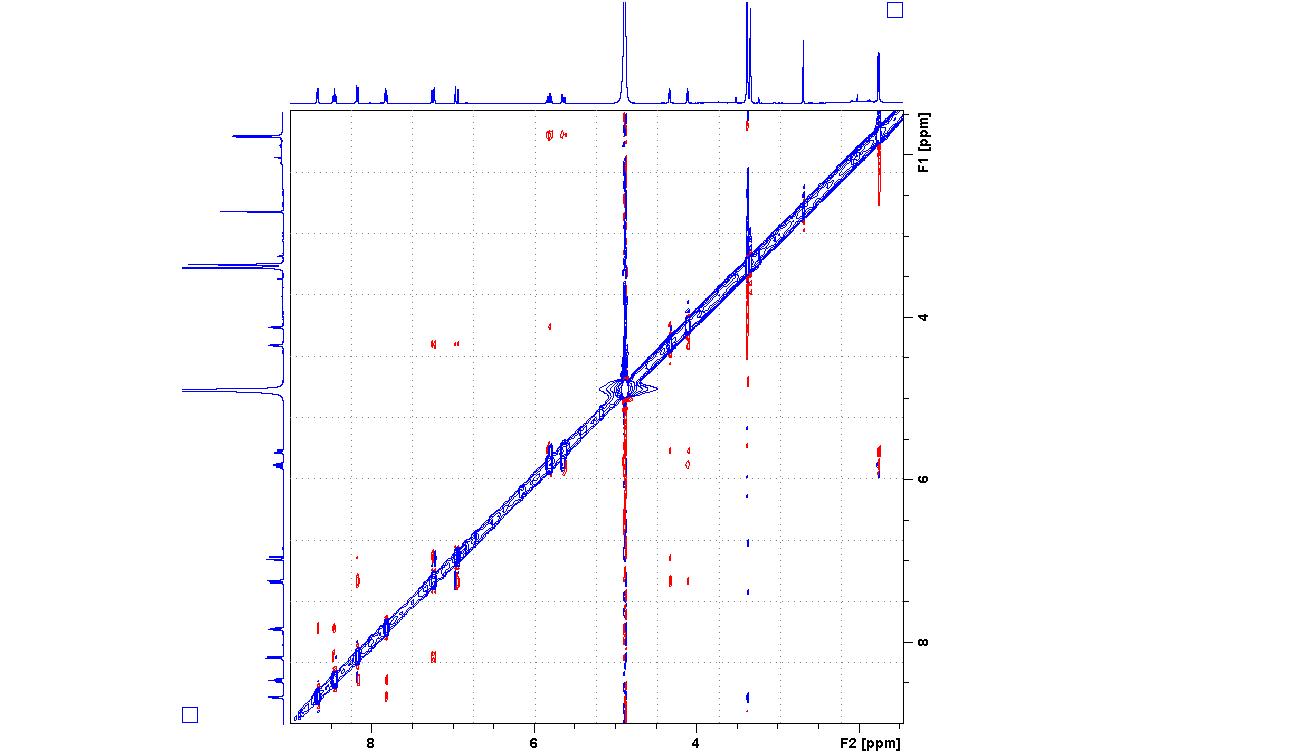


**Figure S39.** NOESY spectrum of ARP DE45


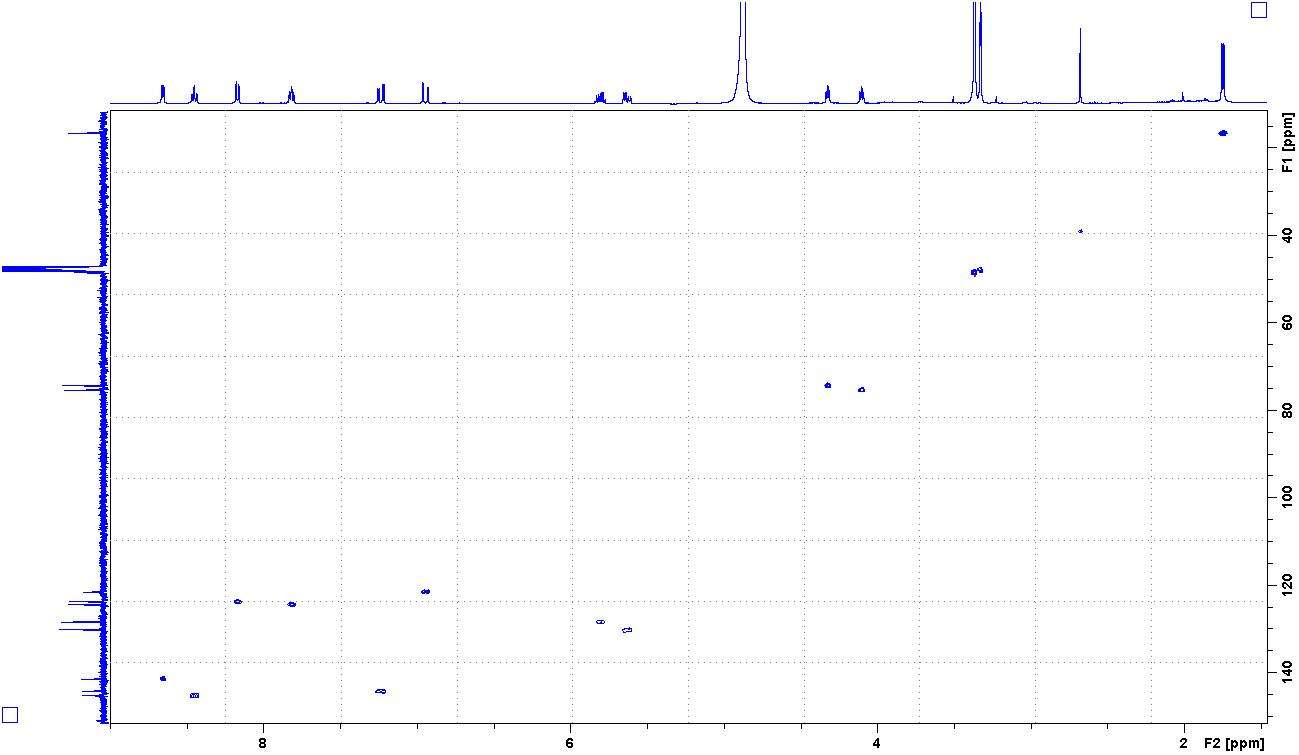


**Figure S40.** Edited HSQC spectrum of ARP DE45


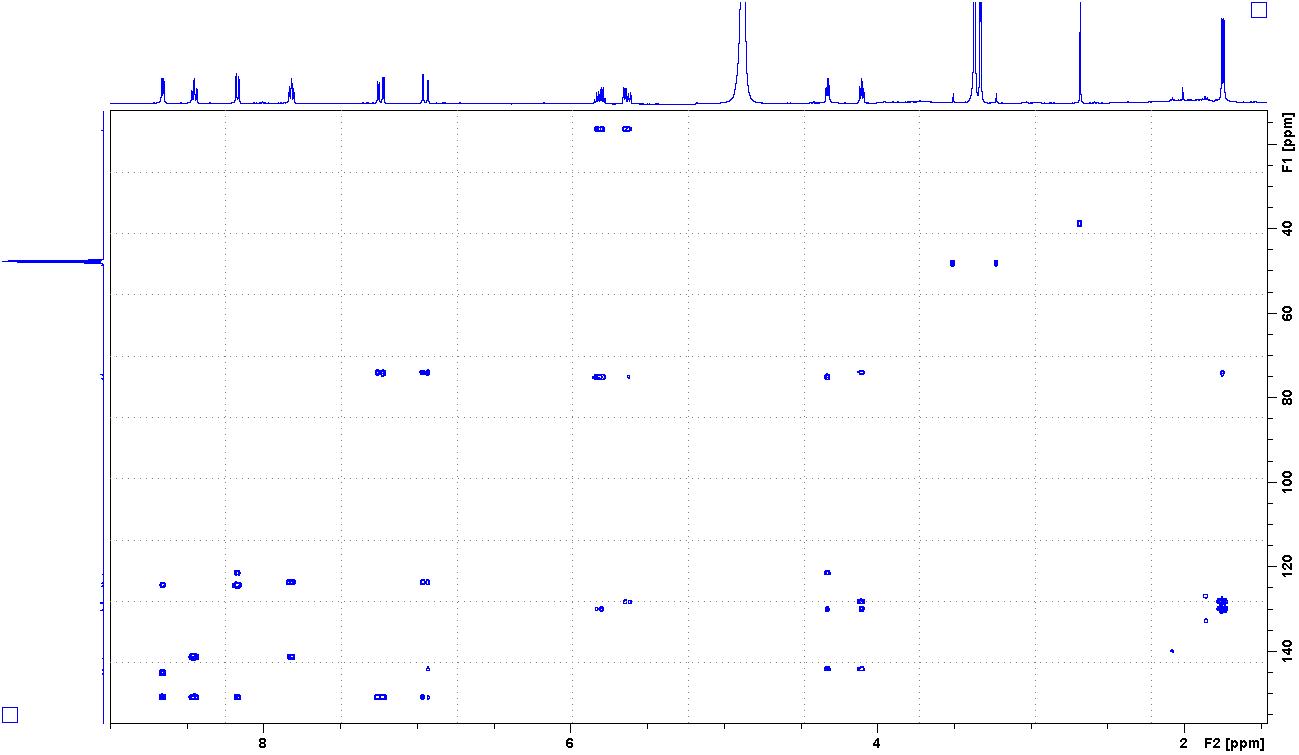


**Figure S41.** HMBC spectrum of ARP DE45

**Figure S42.** UV-vis (DAD) spectrum of ARP DM104

**Figure S43.** ESI-TOF HRMS spectrum of ARP DM104


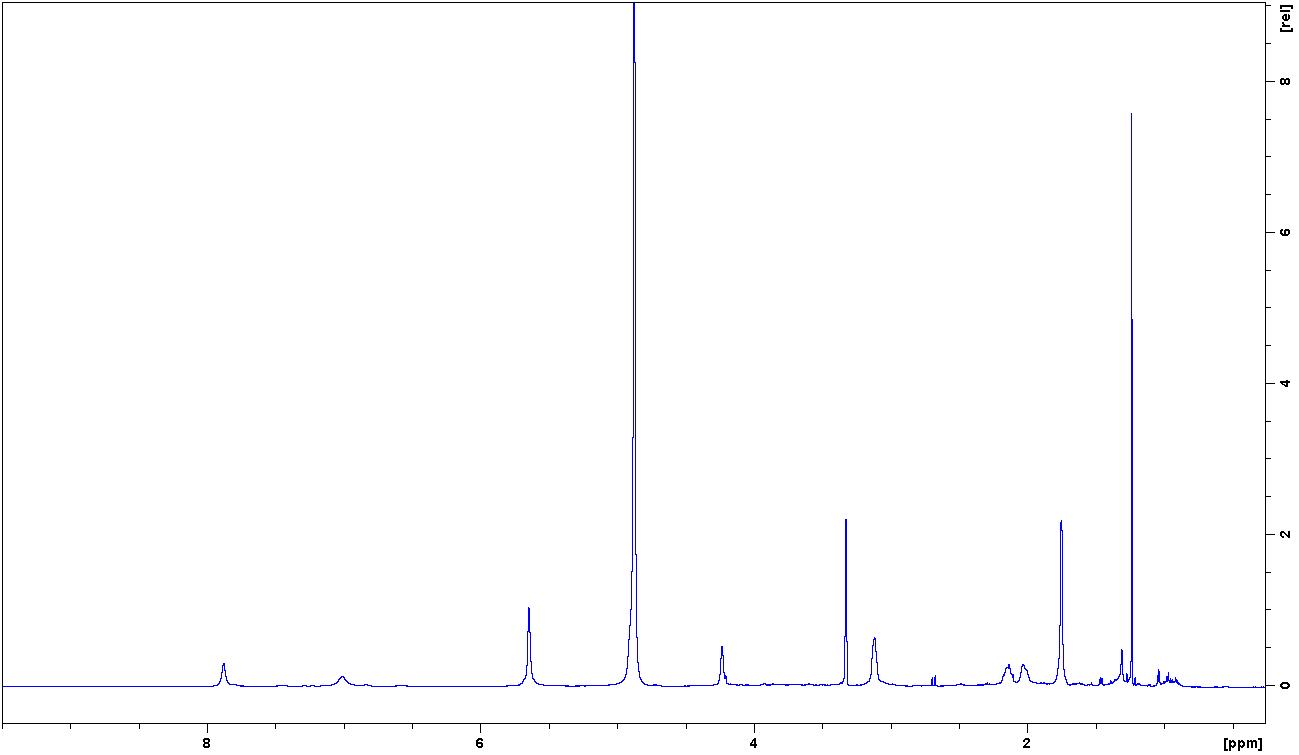


**Figure S44.** ^1^H spectrum of ARP DM104 (CD_3_OD, 24 °C, 500 MHz)


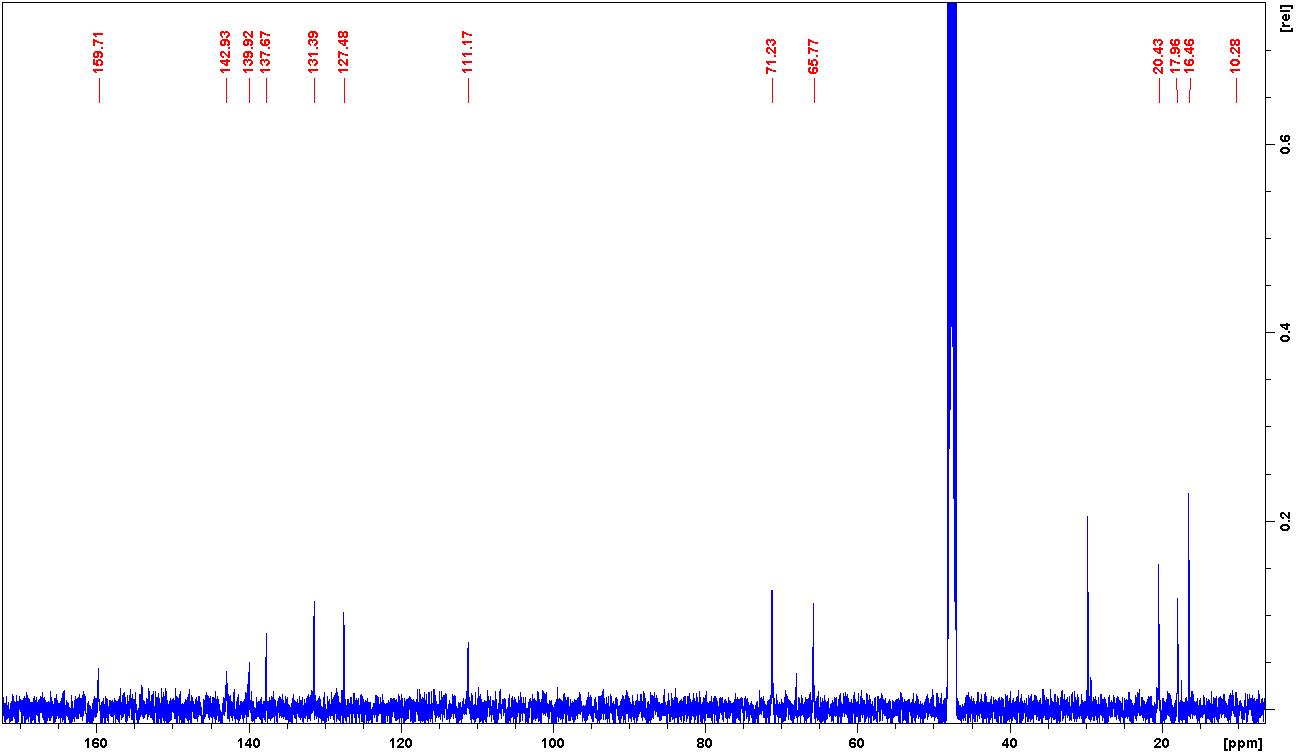


**Figure S45.** ^13^C spectrum of ARP DM104 (CD_3_OD, 24 °C, 125 MHz)


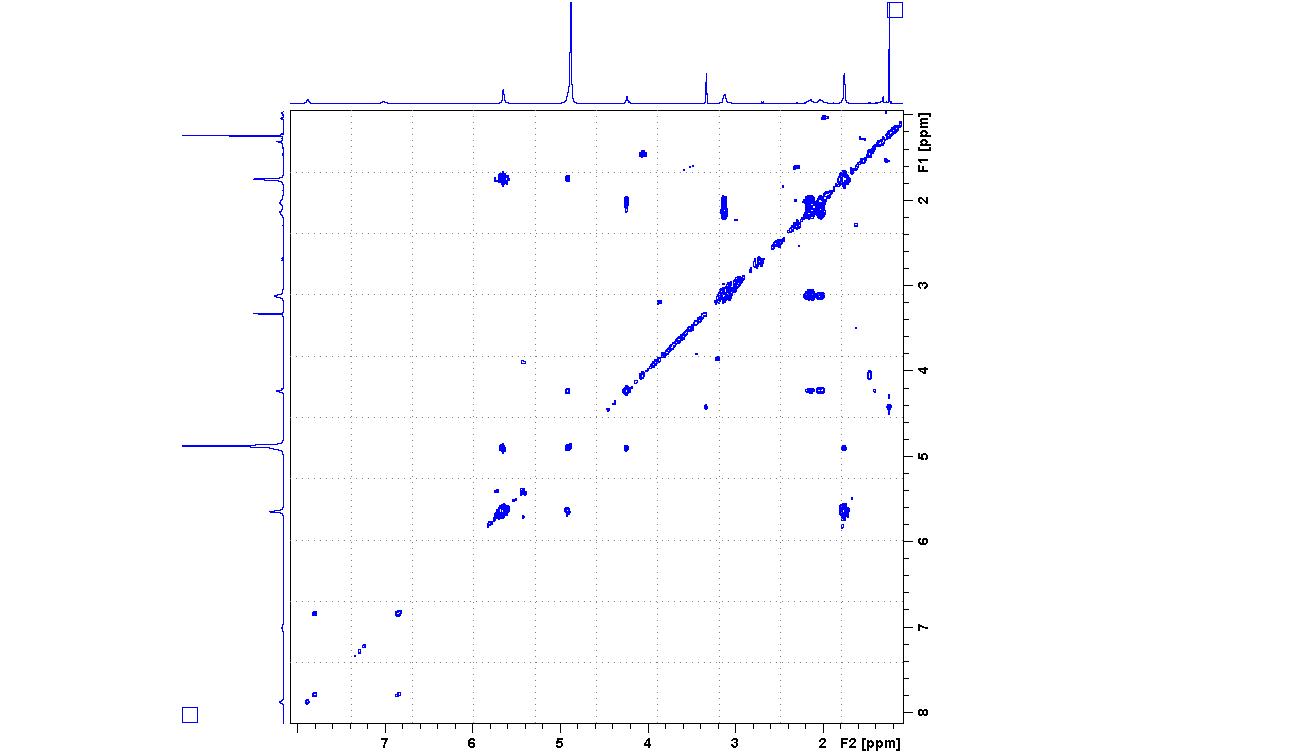


**Figure S46.** COSY spectrum of ARP DE45


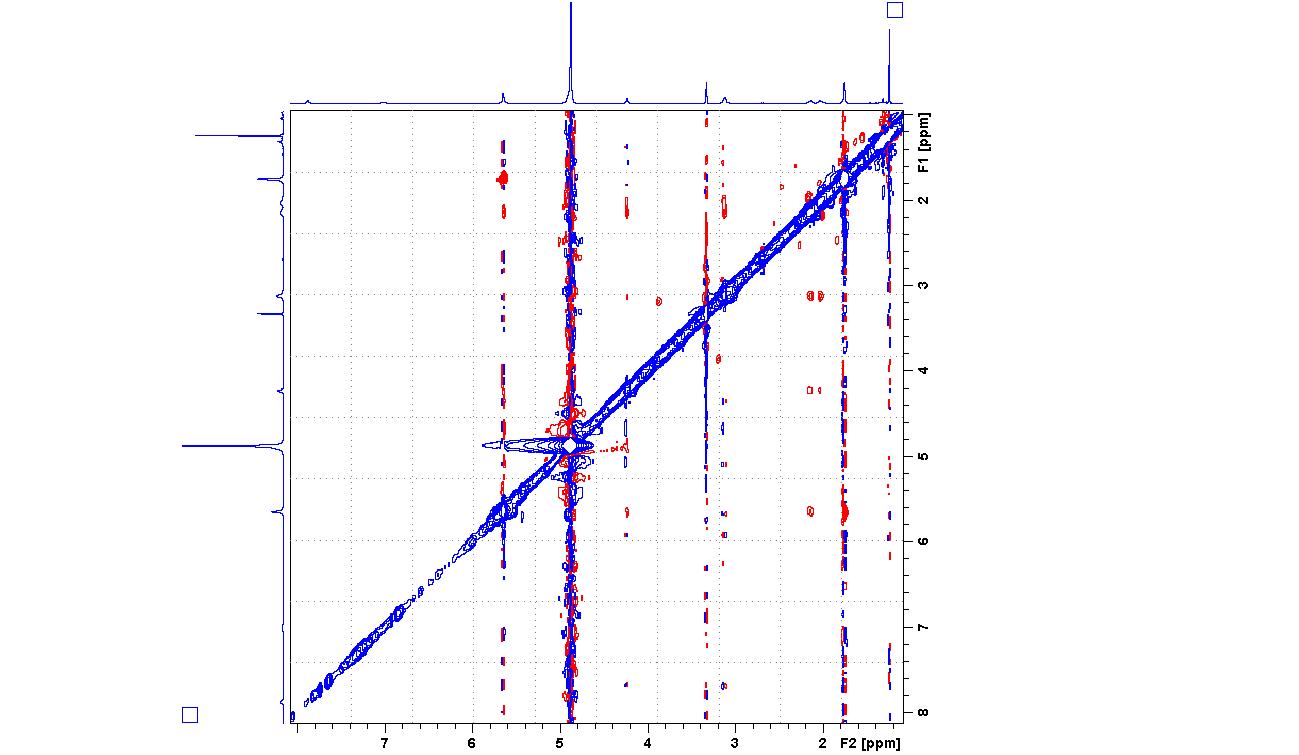


**Figure S47.** NOESY spectrum of ARP DM104


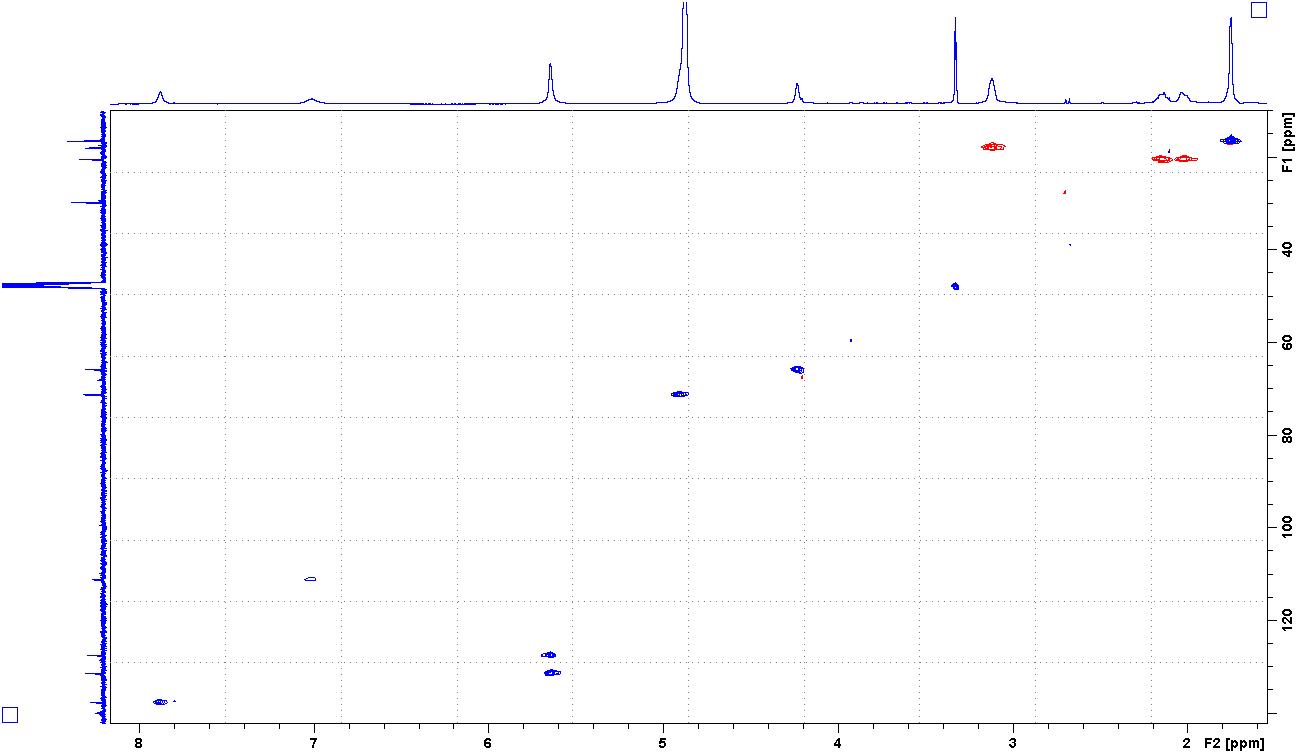


**Figure S48.** Edited HSQC spectrum of ARP DM104


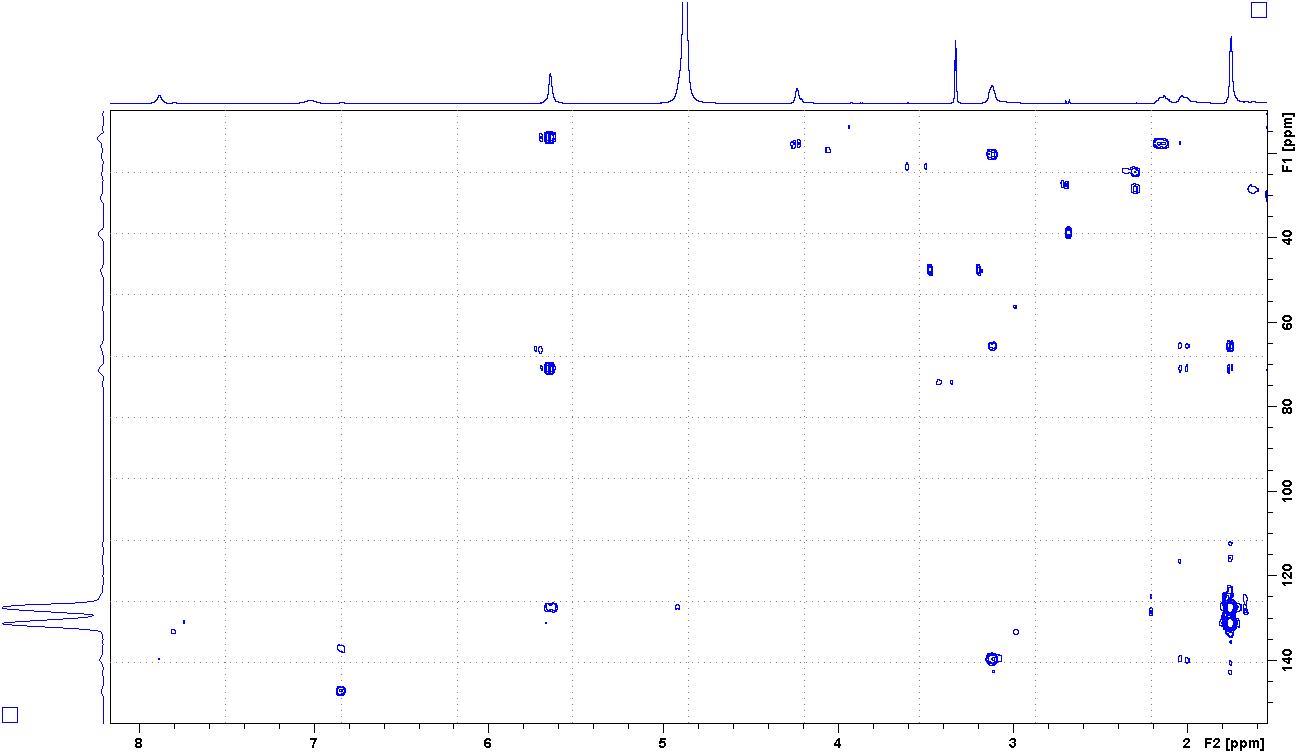


**Figure S49.** HMBC spectrum of ARP DM104
